# Supplementary material for: A fluorogenic probe for granzyme B enables in-biopsy evaluation and screening of response to anticancer immunotherapies
Source: Nat Commun. 2022 May 2;13:2366. doi: 10.1038/s41467-022-29691-w (PMC9061857; doi:10.1038/s41467-022-29691-w)
Supplement: Supplementary file 1 — Supplementary Information [file 41467_2022_29691_MOESM1_ESM.pdf]

<u>Supplementary Information</u>

A fluorogenic probe for granzyme B enables in-biopsy evaluation and screening of response to anticancer immunotherapy

Table of Contents

Supplementary Figures

Supplementary Tables

Supplementary Movies

Supplementary Notes

Supplementary Methods

Supplementary References

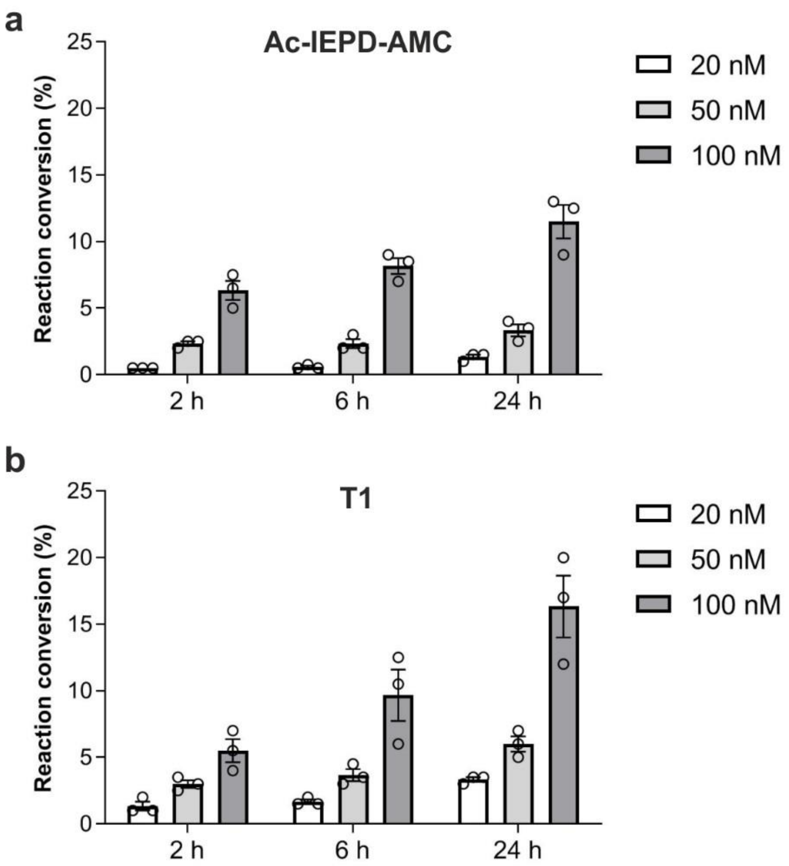

Supplementary Figure 1. Reactivity of fluorogenic substrates against human GzmB and limits of detection. a) Reaction conversion rates (by HPLC-UV detection at 350 nm) for Ac-IEPD-AMC (50 μM) in TRIS buffer (see Methods) at 37 ˚C containing different concentrations of hGzmB. Data presented as mean values±SEM (n=3 independent biological experiments). b) Reaction conversion rates (by HPLC-UV detection at 500 nm) for the tetrapeptide **T1** (25 μM) in TRIS buffer at 37 ˚C containing different concentrations of hGzmB. Data presented as mean values±SEM (3 independent biological experiments). Source data provided as a Source Data file.

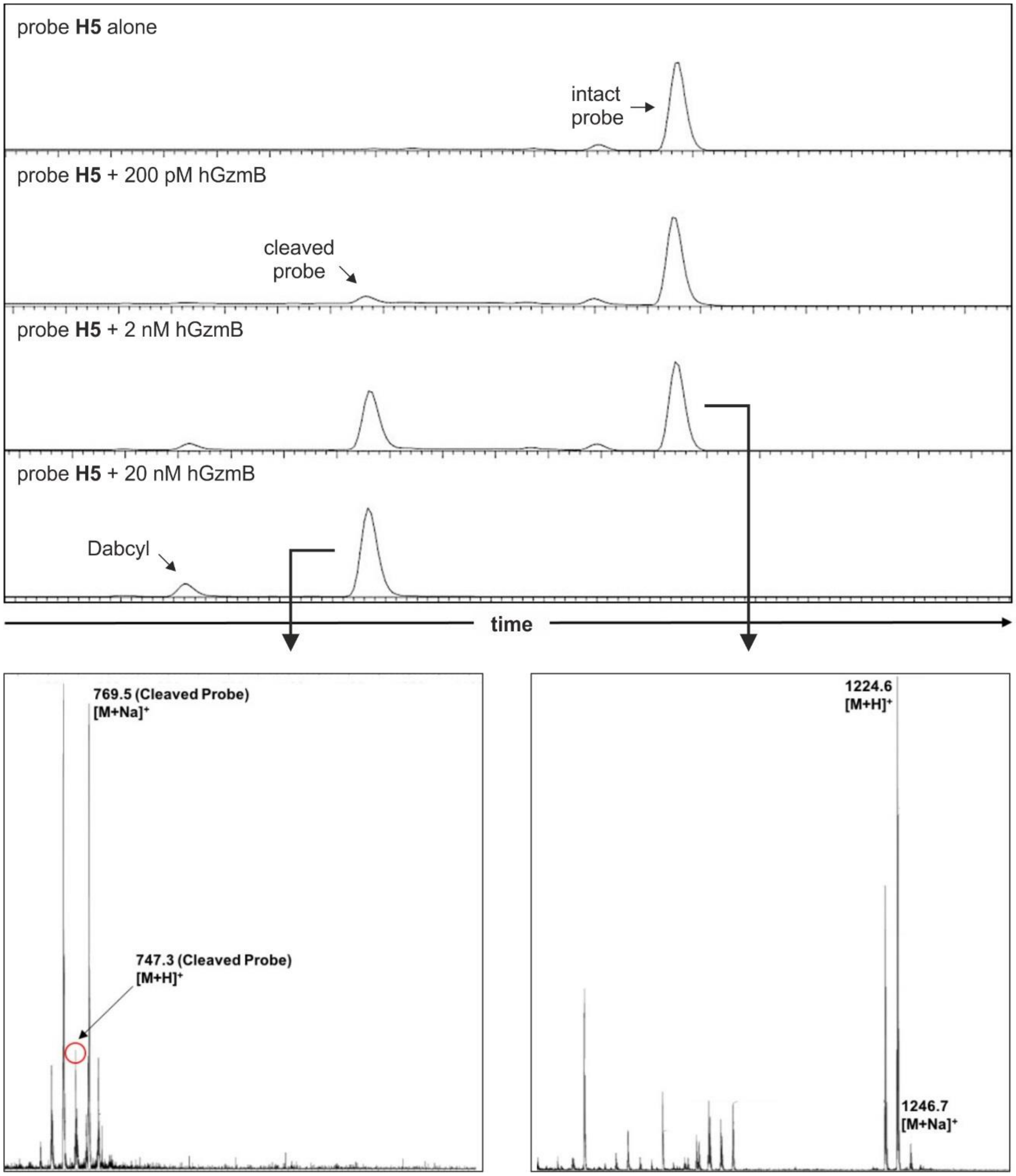

Supplementary Figure 2. HPLC traces of the probe H5 before and after reaction with hGzmB. HPLC chromatogram (UV detection: 500 nm) and mass spectrometry analysis of the probe **H5** (25 μM) before and after reaction with increasing concentrations of hGzmB at 37 ˚C (representative traces from 3 independent experiments). Mcalc. (intact probe): 1246.6 $[M+Na^+]$; Mcalc. (cleaved probe): 747.2 $[M+H^+]$.

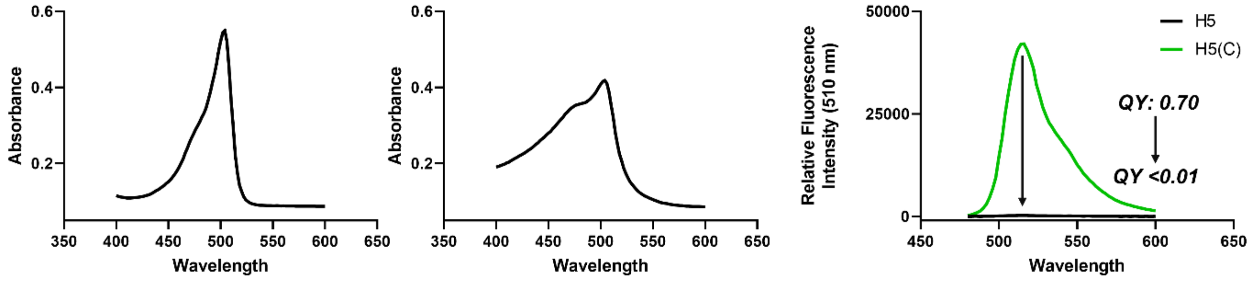

Supplementary Figure 3. Absorbance and emission spectra of probe H5 and probe H5-unquenched. Absorbance spectra of probe **H5-unquenched** (left) and probe **H5** (center), both at 25 μM. Fluorescence emission spectra of both probes (right) and fluorescence quantum yields after excitation at 450 nm. Source data provided as a Source Data file.

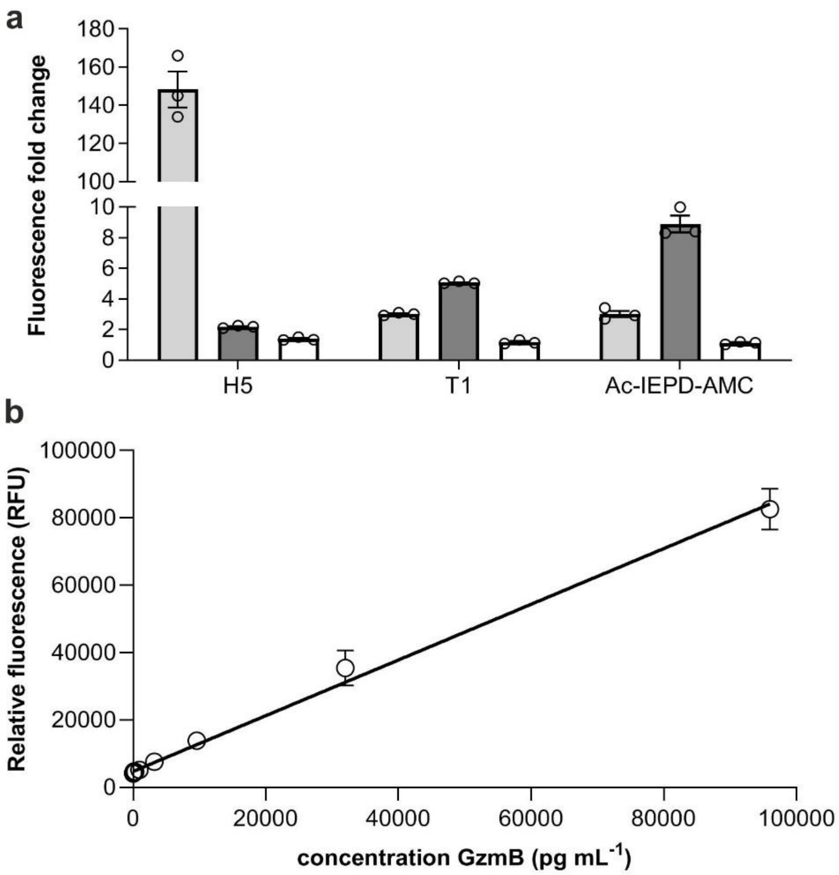

Supplementary Figure 4. Comparative enzyme selectivity and limit of detection for the hexapeptide H5. a) Fluorescence response for the fluorogenic peptides **H5**, **T1** and Ac-IEPD-AMC (all at 25 µM) after incubation for 90 min at 37 °C with hGzmB (light gray bars), human caspase-3 (dark gray bars) and hGzmA (white bars) (all enzymes used at 20 nM). Data presented as mean values±SEM (n=3). Excitation wavelengths: 450 nm (for **H5** and **T1**), 340 nm (for Ac-IEPD-AMC). Emission wavelengths: 510 nm (for **H5** and **T1**), 450 nm (for Ac-IEPD-AMC). b) Limit of detection of hGzmB by fluorescence emission (530 nm) of the hexapeptide **H5** (25 µM) after reaction with increasing amounts of hGzmB (0, 9.6, 32, 96, 320, 960, 3,200, 9,600, 32,000 and 96,000 pg mL^{-1}) at 37 ˚C. Data presented as mean values±SD (2 independent experiments with 3 technical replicates). Source data provided as a Source Data file.

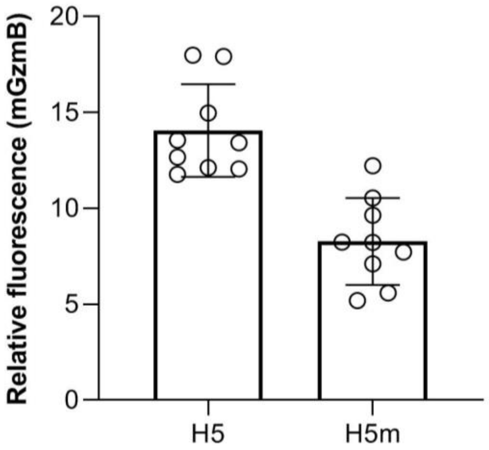

Supplementary Figure 5. **Reactivity of the hexapeptides H5 and H5m against recombinant mouse GzmB.** Fluorescence fold increase for the fluorogenic peptides **H5** and **H5m** (both at 25 μM) after incubation for 90 min at 37 °C with recombinant mouse pro-GzmB (100 nM) after pre-activation with mouse cathepsin C (5.5 μg mL^{-1}) for 4 h. Excitation/emission wavelengths: 450 nm/510 nm. Data presented as mean values±SEM (3 independent experiments with 3 technical replicates). Source data provided as a Source Data file.

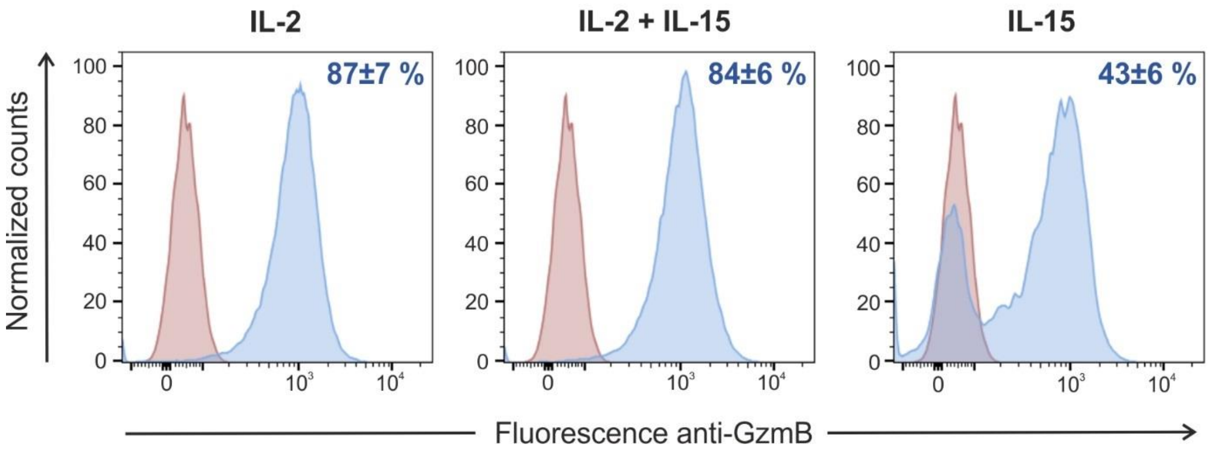

Supplementary Figure 6. Analysis of GzmB expression in CD8+ T cells following cytokine stimulation. Representative histograms (from experiments performed in triplicate) of GzmB expression in murine naïve CD8+ T cells following 2 days of culture with anti-mouse CD3 (2 µg mL^{-1}), anti-mouse CD28 (5 µg mL^{-1}) and different cytokines at the following concentrations: IL-2 (60 U mL^{-1}), IL-15 (60 U mL^{-1}). Cells were incubated with anti-mouse GzmB PE (50 nM) and analysed by flow cytometry in a 5LSR flow cytometer. The histograms show naïve CD8+ T cells in red and cytokine-stimulated cells in blue. The percentage of anti-GzmB+ CD8+ T cells is shown for every condition. Excitation/emission wavelengths for anti-mouse GzmB PE: 561 nm/582 nm.

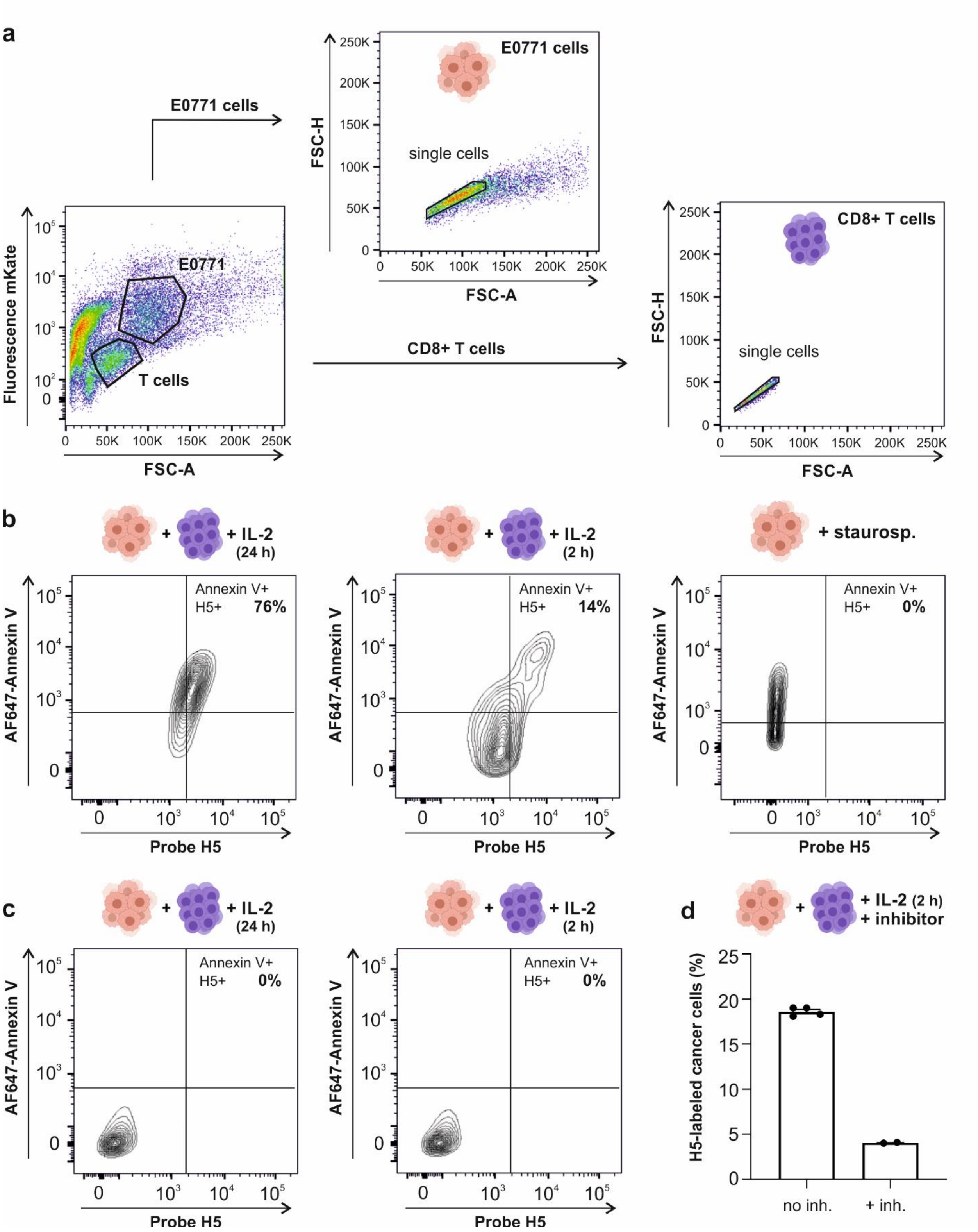

Supplementary Figure 7. **Gating strategy and flow cytometry contour plots for co-cultures of mouse CD8+ T cells and E0771 cells**. a) Gating strategy for co-cultures of

mouse CD8+ T cells and mKate-E0771 cells. b) Representative flow cytometry contour plots of E0771 cells in: left) co-cultures of E0771 cells (50,000 cells well^{-1}) and activated (IL-2 for 24 h) murine CD8+ T cells (200,000 cells well^{-1}), center) co-cultures of E0771 cells (50,000 cells well^{-1}) and non-activated (IL-2 for 2 h) murine CD8+ T cells (200,000 cells well^{-1}), right) E0771 alone cells treated with staurosporine (1 μM, 1 h). c) Representative flow cytometry contour plots of CD8+ T cells in: left) co-cultures of E0771 cells (50,000 cells well^{-1}) and activated (IL-2 for 24 h) murine CD8+ T cells (200,000 cells well^{-1}), center) co-cultures of E0771 cells (50,000 cells well^{-1}) and non-activated (IL-2 for 2 h) murine CD8+ T cells (200,000 cells well^{-1}). All cultures in b) and c) were equally stained with probe **H5** (5 μM) and AF647-Annexin V (10 nM). d) Fluorescence intensity from **H5**-labeled E0771 cancer cells following co-culture with IL-2 activated CD8+ T cells with and without preincubation of the GzmB inhibitor Ac-IEPD-CHO. Data presented as mean values±SD. Excitation/emission wavelengths: 488 nm/525 nm (for probe **H5**), 633/670 nm (for AF647-Annexin V). Representative plots from independent experiments performed in triplicate. Source data in d provided as a Source Data file.

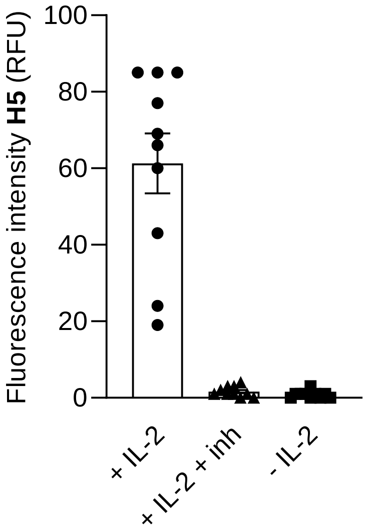

Supplementary Figure 8. Quantification of the fluorescence signals of probe H5 in co-cultures of T cells and mKate-expressing E0771 cancer cells. Image analysis (representative images shown in Figure 2d) of co-cultures that had been treated or not with IL-2 and the GzmB inhibitor Ac-IEPD-CHO, followed by staining with compound **H5** (25 μM). Data presented as mean values±SEM (10 different regions of interest for each of the conditions). Source data provided as a Source Data file.

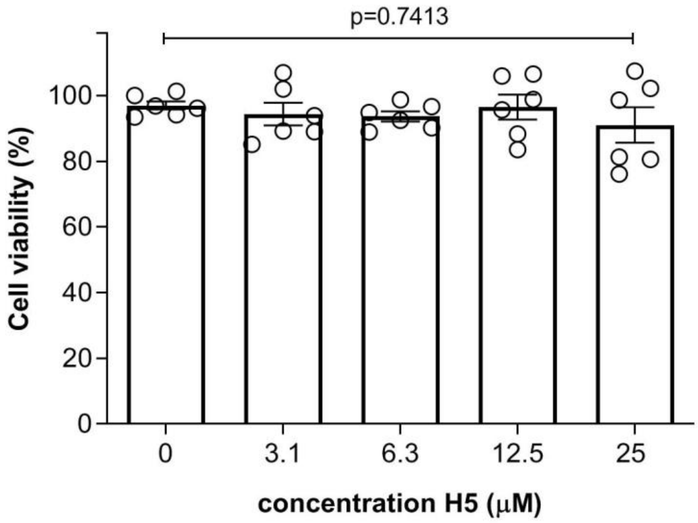

Supplementary Figure 9. **Cell viability assays in mKate-E0771 cancer cells**. mKate-E0771 cells were plated in 96-well plates (50,000 cells well^{-1}) and incubated at the indicated concentrations of probe **H5** for 1 h at 37 °C. Cell viability was determined using a commercially available MTT kit (Invitrogen) with values normalized to untreated cells. Data presented as mean values±SEM (2 independent experiments with 3 replicates for each). P values were obtained from ONE-ANOVA tests with multiple comparisons. Source data provided as a Source Data file.

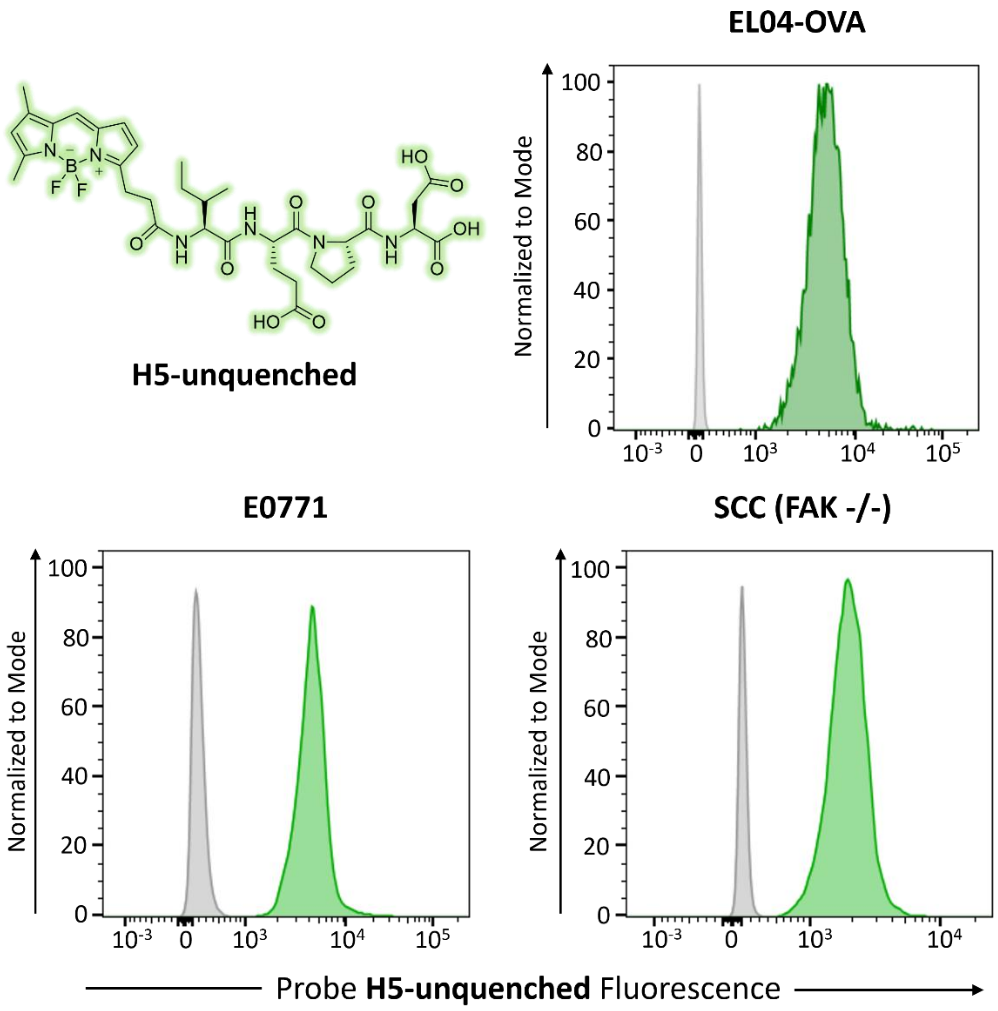

Supplementary Figure 10. Cell permeability analysis of the compound H5-unquenched. Chemical structure of the 'always-on' compound **H5-unquenched** and representative fluorescence histograms of multiple cancer cell lines (2.5×10^5 cells mL^{-1}) after incubation with compound **H5-unquenched** (25 μM) for 30 min at r.t. Cells were washed twice with PBS before analysis by flow cytometry in a 5LSR flow cytometer. The histograms show unstained cells in grey and treated cells in green. Excitation/emission wavelengths: 488 nm/525 nm. Representative plots from independent experiments performed in triplicate.

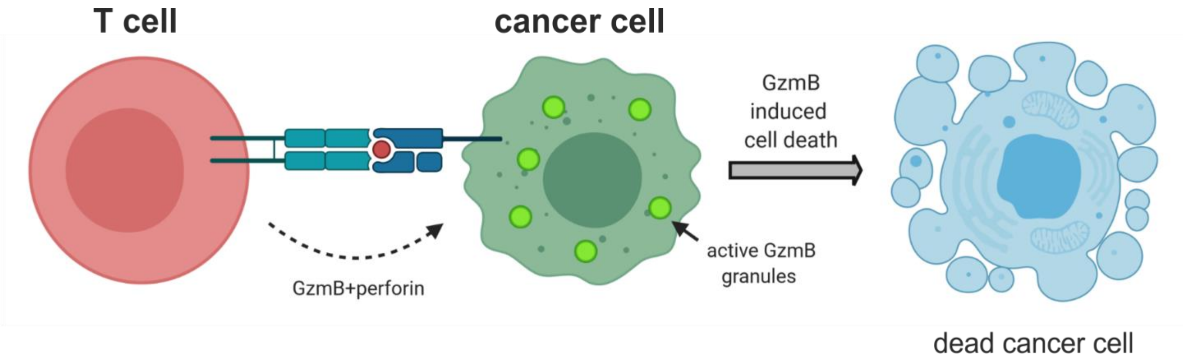

Supplementary Figure 11. Schematic representation of CD8$^+$ T cell GzmB-mediated cytotoxicity against cancer cells. The illustration represents the antigen recognition between a CD8$^+$ T cell and the MHC-I complex on a target cancer cell. Perforin and GzmB are released along the immunological synapse whereby perforin facilitates the entry of GzmB into the cytosol of the target cell. Upon activation, GzmB initiates apoptosis via caspase and caspase-independent pathways.

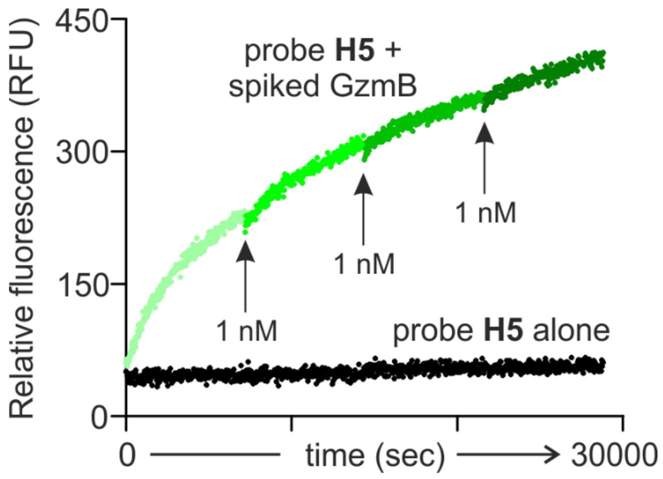

Supplementary Figure 12. Fluorescence increase of H5 with incremental additions of recombinant human GzmB Longitudinal fluorescence emission of probe **H5** (1 nM) upon spiking hGzmB at time 0 and at different timepoints highlighted by the arrows (1 nM increments of hGzmB) (n=3). Source data provided as a Source Data file.

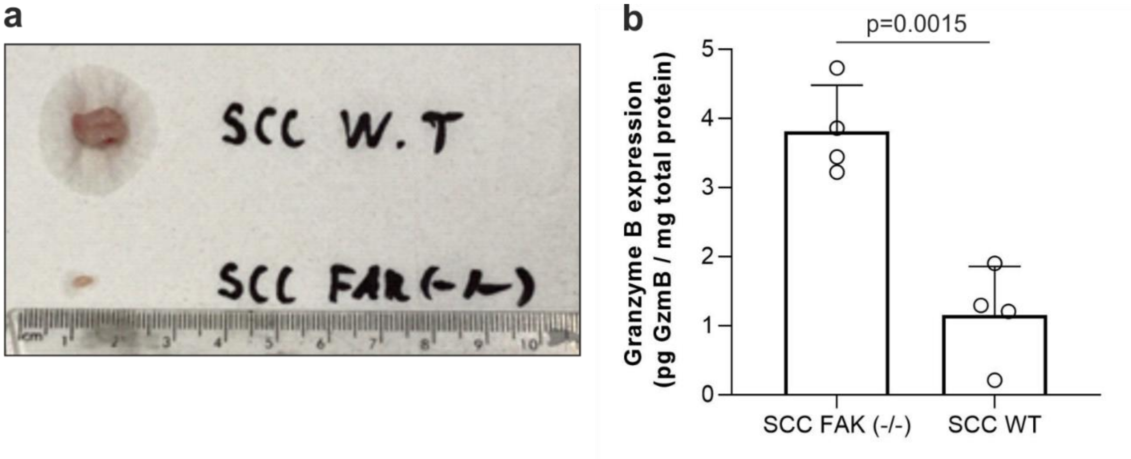

Supplementary Figure 13. Quantification of GzmB levels in SCC7.1 and SCC7.1 FAK-/- tumors. a) Representative picture of harvested SCC7.1 and SCC7.1 FAK-/- tumors on day 14 post-injection of cancer cells. b) Quantification of GzmB levels in both tumous by ELISA after tissue disaggregation and protein extraction. GzmB levels were normalized to total protein amount as measured by the BCA method. Data presented as mean values±SD (n=4). P values were obtained from two-tailed *t* tests. Source data in b provided as a Source Data file.

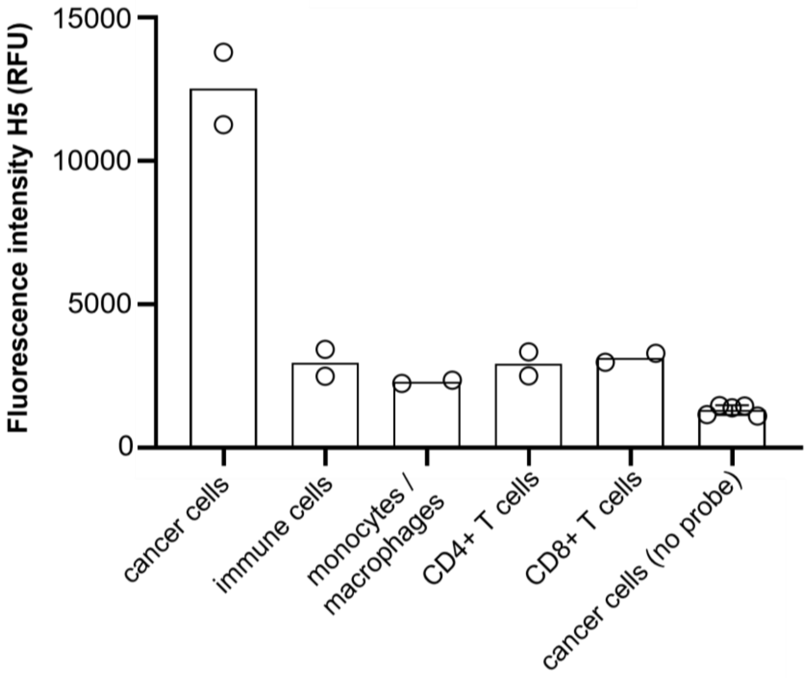

Supplementary Figure 14. Mean fluorescence intensities of different cell populations in SCC (-/-) tumor digests after incubation with probe H5. Fluorescence emission in multiple cell subsets as gated in Supplementary Figure 14. Data presented as mean values±SEM (2 independent experiments). Source data provided as a Source Data file.

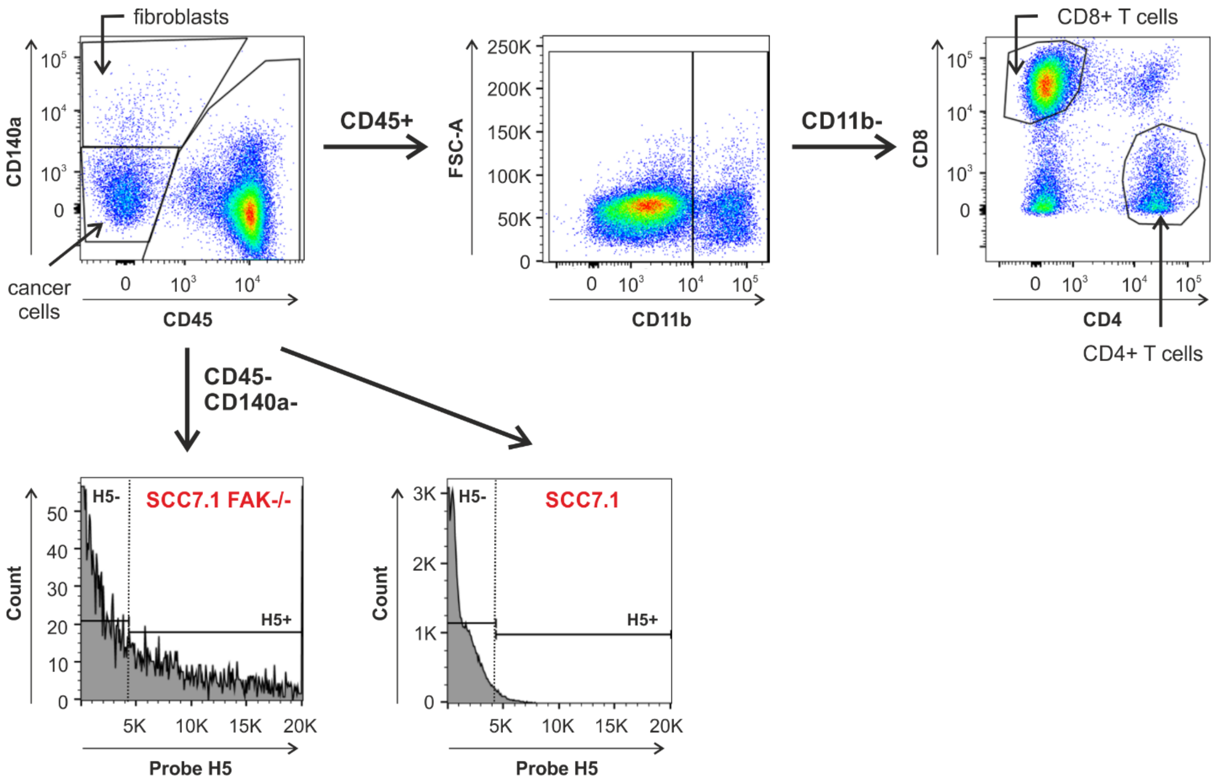

Supplementary Figure 15. Gating strategy for assessing the probe H5 in SCC7.1 and SCC7.1 FAK-/- tumors. All cells were gated on singlets and live cells (Zombie NIR viability dye, 1:1000 dilution). Wild-type SCC and SCC FAK-/- were gated as CD45− CD140a− (AF700 & BV650, respectively). All non-cancerous and non-fibrotic cells were gated on CD45+ (AF700). Monocytes were gated on CD11b+ (PerCP-Cy5.5) whilst the remaining cells were classified as CD8+ T cells (PE), CD4+ T cells (eFluor450) or immune cells (CD4−CD8− or CD4+CD8+).

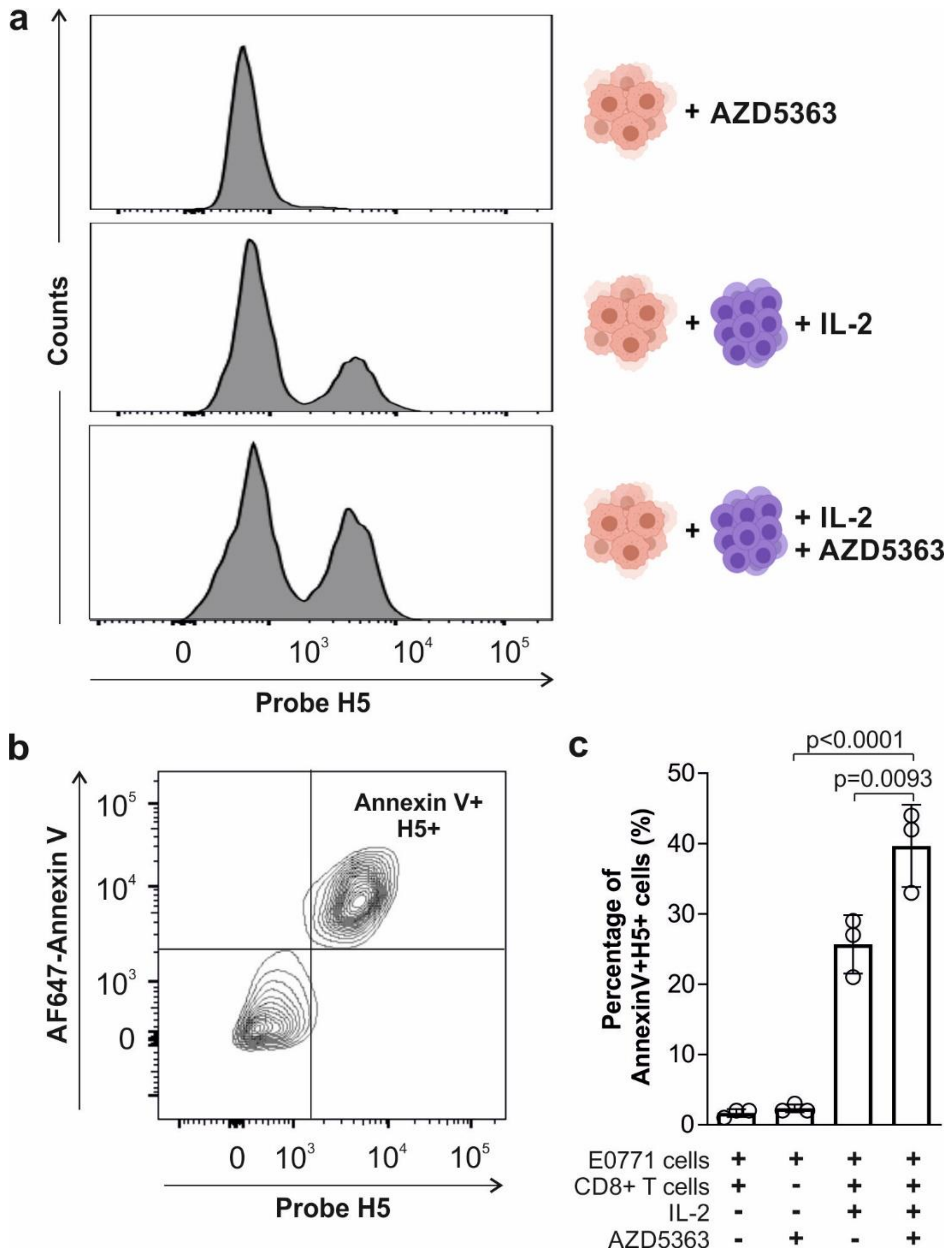

Supplementary Figure 16. Probe H5 detects CD8+ T cell mediated killing of cancer cells induced by IL-2 and AZD5363. a) Representative histograms of probe **H5** staining (from 3 independent experiments) in E0771 cells alone (50,000 cells well^{-1}) or in co-culture with CD8+ T cells (200,000 cells well^{-1}) and treatment of AZD5363 (1 μM). IL-2 concentration: 1,000 U mL^{-1}. Excitation/emission wavelengths: 488 nm/525 nm. b) Representative flow cytometry contour plots (from 3 independent experiments; gating

strategy shown in Supplementary Figure 7) of E0771 cells (50,000 cells $well^{-1}$) after co-culture with murine CD8+ T cells (200,000 cells $well^{-1}$) and dual treatment with IL-2 (200 U mL^{-1}) and AZD5363 (1 μM). Co-cultures were stained with probe **H5** (5 μM) and AF647-Annexin V (10 nM). Excitation/emission wavelengths: 488 nm/525 nm (for probe **H5**), 633/670 nm (for AF647-Annexin V). c) Percentages of E0771 cells that were double-stained with probe **H5** and AF647-Annexin V under the experimental conditions described in a). Data presented as mean values±SEM (n=3). P values ($p<0.001$ and $p=0.0093$) were obtained from ONE-ANOVA tests with multiple comparisons. Source data in c provided as a Source Data file.

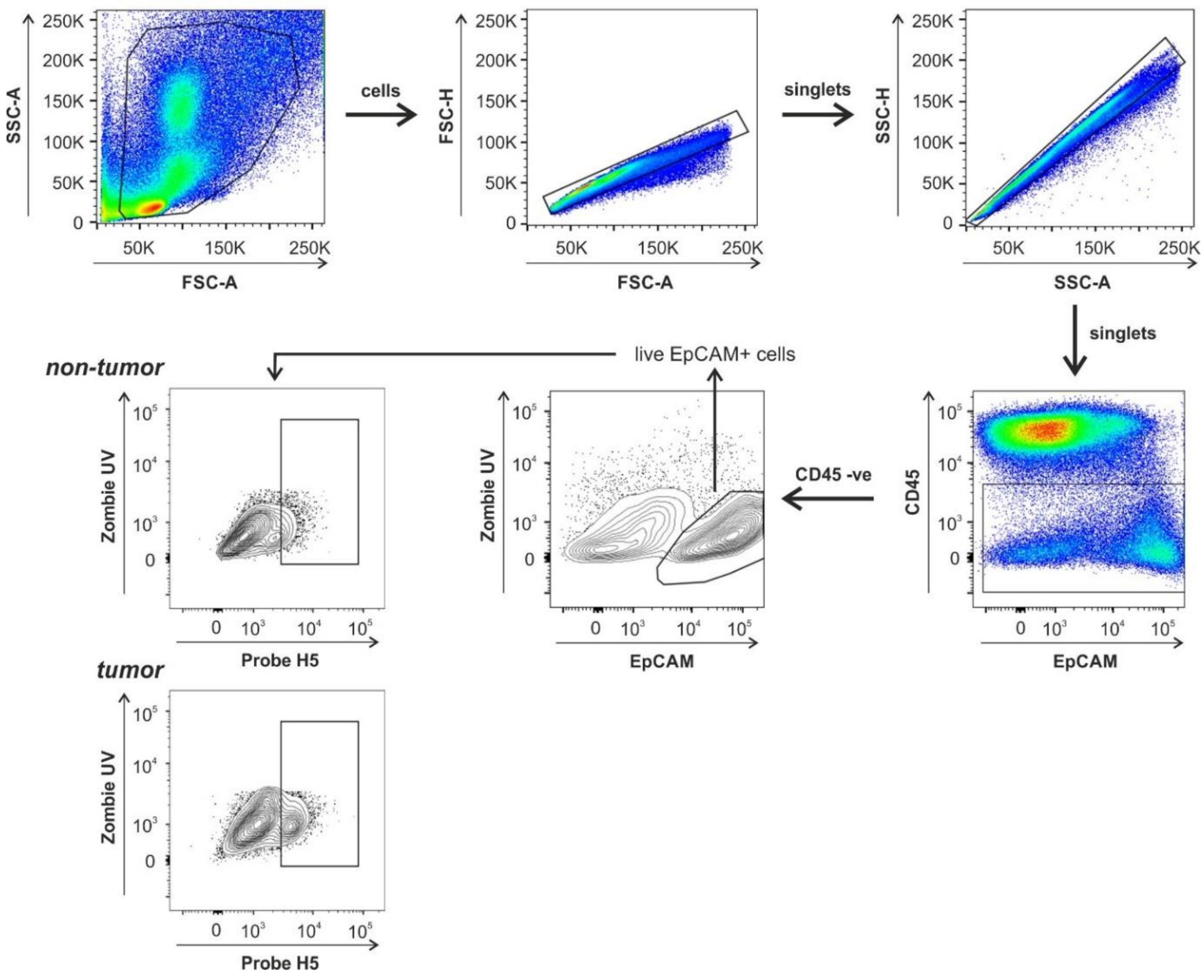

Supplementary Figure 17. **Gating strategy for assessing the probe H5 in human lung tissue.** All cells were gated to exclude debris and gated on singlets. Leukocytes were excluded on the basis of CD45 positivity, and live EpCAM positive cells were selected by excluding cells stained positive with the Zombie UV viability dye (1:1000 dilution). Probe **H5** positivity in the live EpCAM+ population in non-tumor and tumor tissues was assessed against cells stained in the absence of probe **H5** giving a background of <1% positivity.

Supplementary Table 1. Chemical structures and reactivity of FRET-based tetrapeptides against human GzmB.

Code	Structure	Linker	Quencher	HPLC conversion (20 nM hGzmB, 2 h)	HPLC conversion (100 nM hGzmB, 24 h)
**T1**	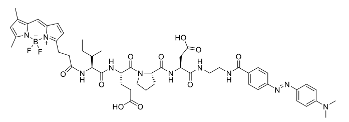	C2	Dabcyl	3%	14%
**T2**	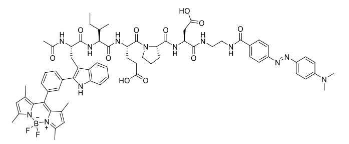	C2	Dabcyl	<1%	4%
**T3**	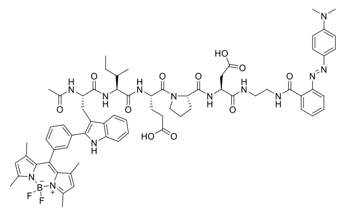	C2	Methyl Red	<1%	1%
**T4**	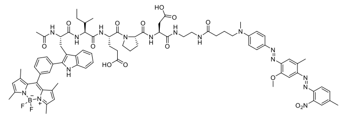	C2	BHQ-1	2%	2%
**T5**	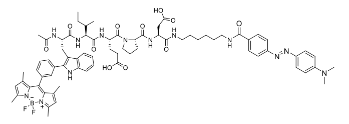	C6	Dabcyl	<1%	5%

Supplementary Table 2. Sequences and reactivity of FRET-based hexapeptides against hGzmB.

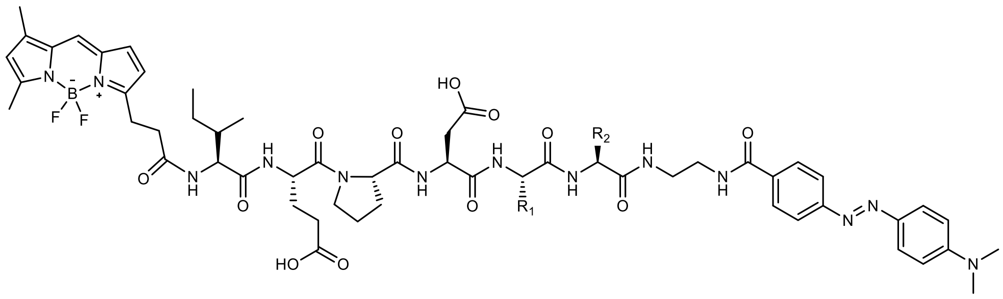

Code	Peptide sequence	HPLC conversion (20 nM hGzmB, 2 h)
**H1**	Ile-Glu-Pro-Asp-Ala-Gly	75%
**H2**	Ile-Glu-Pro-Asp-Ser-Gly	80%
**H3**	Ile-Glu-Pro-Asp-Ser-Leu	80%
**H4**	Ile-Glu-Pro-Asp-Trp-Leu	82%
**H5**	Ile-Glu-Pro-Asp-Ala-Leu	100%
**H6**	Ile-Glu-Pro-Asp-Trp-Arg	40%
**H7**	Ile-Glu-Pro-Asp-Arg-Leu	60%
**H5m**	Ile-Glu-Phe-Asp-Ala-Leu	70%

Supplementary Table 3. Chemical characterization (HPLC purity and HRMS) for all synthesized probes.

Code	HPLC purity	$M_{calc.}$	$M_{exp.}$
**T1**	98%	1040.4987 [M+H$^+$]	1040.4972
**T2**	97%	1316.6231 [M+H$^+$]	1316.6237
**T3**	96%	1316.6231 [M+H$^+$]	1316.6222
**T4**	98%	1551.7120 [M+H$^+$]	1551.7105
**T5**	99%	1372.6834 [M+H$^+$]	1372.6820
**H1**	97%	1190.5378 [M+Na$^+$]	1190.5377
**H2**	94%	1206.5308 [M+Na$^+$]	1206.5326
**H3**	96%	1240.6129 [M+H$^+$]	1240.6132
**H4**	93%	1339.6605 [M+H$^+$]	1339.6605
**H5**	96%	1246.5989 [M+Na$^+$]	1246.6003
**H6**	95%	1382.6771 [M+H$^+$]	1382.6776
**H7**	99%	1309.6819 [M+H$^+$]	1309.6823
**H5m**	99%	1274.6343 [M+H$^+$]	1274.6339
**H5-unquenched**	98%	746.3253 [M$^+$]	746.3260

Supplementary Table 4. Kinetic values determined for hexapeptides **H2-H5** against recombinant human GzmB.

**Probe**	**Sequence**	**k_{cat}**	**k_{cat} / K_M**
**H2**	IEPDSG	11.2	0.4×10^6
**H3**	IEPDSL	23.6	0.9×10^6
**H4**	IEPDWL	28.2	1.8×10^6
**H5**	IEPDAL	117.0	1.2×10^7

Supplementary Table 5. Screened small molecule drugs and working concentrations.

Code	Compound	Conc. (μM)	Code	Compound	Conc. (μM)
**C1**	Dinaciclib (CDK inhibitor)	1	**C23**	Olaparib (PARP inhibitor)	1
**C2**	Seliciclib (CDK inhibitor)	1	**C24**	Veliparib (PARP inhibitor)	1
**C3**	Genistein (tyrosine kinase inhibitor)	10	**C25**	Cytochalasin B (actin polymerization inhibitor)	3
**C4**	Trametinib (MEK inhibitor)	0.1	**C26**	Latrunculin B (actin polymerization inhibitor)	1
**C5**	AZD5363 (AKT inhibitor)	1	**C27**	*N*-acetyl-leucyl-leucyl-norleucinal (calpain inhibitor)	1
**C6**	Saracatinib (Src/Abl inhibitor)	1	**C28**	ZVAD (caspase inhibitor)	1
**C7**	Dasatinib (Src/Abl inhibitor)	0.1	**C29**	*N*-acetyl-leucyl-leucyl-methioninal (calpain inhibitor)	1
**C8**	ZM447439 (Aurora inhibitor)	1	**C30**	Lactacystin (proteasome inhibitor)	1
**C9**	Y27632 (ROCK inhibitor)	1	**C31**	Valproic Acid (histone deacetylase inhibitor)	10
**C10**	Paclitaxel (microtubule inhibitor)	0.3	**C32**	Trichostatin A (histone deacetylase inhibitor)	1
**C11**	Docetaxel (microtubule inhibitor)	1	**C33**	SAHA (histone deacetylase inhibitor)	10
**C12**	ARQ621 (Eg5 inhibitor)	10	**C34**	Panobinostat (histone deacetylase inhibitor)	0.1
**C13**	Epothilone B (microtubule inhibitor)	1	**C35**	Emetine (protein synthesis inhibitor)	1
**C14**	Barasertib (Aurora kinase B inhibitor)	1	**C36**	AZD2014 (mTOR inhibitor)	1
**C15**	Nocodazole (microtubule inhibitor)	0.3	**C37**	Brefeldin A (Golgi apparatus breakdown)	1
**C16**	Methotrexate (DHFR inhibitor)	10	**C38**	CA074Me (cathepsin B inhibitor)	3
**C17**	Aphidicolin (DNA polymerase inhibitor)	1	**C39**	Simvastatin (HMG-CoA reductase inhibitor)	1
**C18**	Mitomycin C (DNA crosslinker)	1	**C40**	Lovastatin (HMG-CoA reductase inhibitor)	1
**C19**	Floxuridine (TS synthetase inhibitor)	10	**C41**	Marimastat (MMP inhibitor)	3
**C20**	Temozolomide (DNA alkylating)	1	**C42**	Pepstatin (Aspartyl protease inhibitor)	10
**C21**	Camptothecin (topoisomerase inhibitor)	1	**C43**	Leupeptin (Ser/Cys protease inhibitor)	1
**C22**	SN38 (topoisomerase inhibitor)	1	**C44**	Aprotinin (Trypsin inhibitor)	10

Supplementary Movie 1. Real-time imaging of probe H5 in co-cultures of OT-I CD8+ T cells and OVA-EL4 cancer cells. Time-course widefield microscope images of OT-I CD8+ T cells (counterstained with Cell Tracker Orange, red) killing OVA-EL4 cancer cells in the presence of probe **H5** (10 μM, green) and the dead marker Sytox Blue (1 μM, blue). Scale bar: 10 μm.

Supplementary Movie 2. Brightfield and fluorescence time-lapse imaging of probe H5 in co-cultures of OT-I CD8+ T cells and OVA-EL4 cancer cells. Time-course widefield microscope images of OT-I CD8+ T cells (counterstained with Cell Tracker Orange, red) killing OVA-EL4 cancer cells in the presence of probe **H5** (10 μM, green). Scale bar: 5 μm.

Supplementary Note 1

All peptide conjugates were synthesized utilizing solid-phase peptide synthesis using Fmoc/^{t}Bu strategy on a 2-chlorotrityl chloride polystyrene resin. Resin loading was performed with the appropriate Fmoc protected amino acid (1.4 eq) and DIPEA (10 eq) in DCM for 1 h at r.t. Following this, the remaining 2-chlorotrityl groups were capped with MeOH (0.8 µL per mg of resin). Fmoc removal was performed with piperidine (20% in DMF) and Oxyma (1.0 M) to minimise aspartimide formation under 3×5 min cycles. Amino acids were coupled using an excess of Fmoc-protected amino acid (4 eq), COMU (4 eq), Oxyma (4 eq) and DIPEA (8 eq) in DMF for 1.5 h at r.t. Completion of the coupling was monitored by the Kaiser test (and chloranil test after coupling proline). The side chains of aspartic acid and glutamic acid were protected with OBzl groups and the arginine side chain was protected with a NO_2 group, because they can be removed under mild Pd-catalyzed hydrogenation tolerated by the acid-labile BODIPY-FL fluorophore. Fluorophore coupling was performed in solid-phase using BODIPY-FL (1.1 eq), COMU (1.15 eq), Oxyma (1.15 eq) and DIPEA (3 eq) in DMF for 1.5 h at r.t. Resin cleavage was performed by treating the resin with TFA (1% in DCM) in 5×1 min cycles. The combined solutions were then poured over DCM and evaporated under reduced pressure. Purification was performed by semi-preparative HPLC, and the pure fluorescent peptides were then coupled to a Cbz-protected 1,2-diaminoethane. A catalytic hydrogenation was then performed to remove all protecting groups allowing the introduction of the quencher, Dabcyl, at the terminal amine of the linker via an NHS mediated coupling. Final peptides were isolated by semi-preparative HPLC in purities >95%.

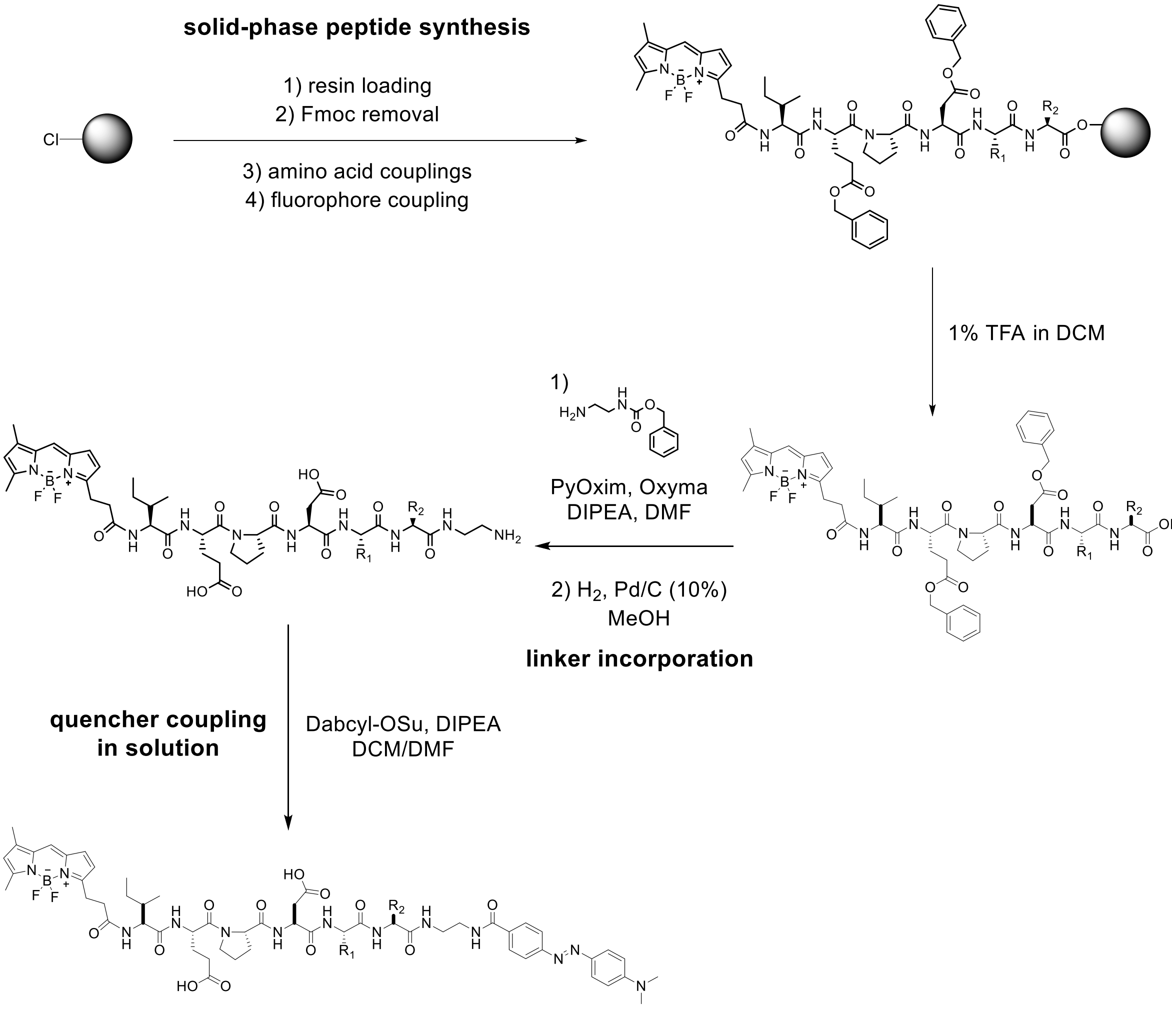

Supplementary Figure 18. **Synthetic route to hexapeptide probes.** Schematic representation of the synthesis of the hexapeptide probes from the 2-chlorotrityl chloride linker on PS resin to the final compound matching the synthetic protocol in Supplementary Note 1.

Supplementary Note 2

Increased fluorescence signals (compared to IL-2 only controls) were observed for different combinations of IL-2 with small molecules. Among these, we identified compounds with different mechanisms of action. AZD5363 (**C5**) is a protein kinase AKT inhibitor; docetaxel (**C11**), ARQ-621 (**C12**) and epothilone B (**C13**) are direct inhibitors of microtubule function; mitomycin C (**C18**) and temozolomide (**C20**) are DNA alkylating agents and lactacystin (**C30**) is an irreversible proteasome inhibitor. Some of these compounds are already approved for medical use as anti-cancer drugs. Docetaxel (Taxotere®), mitomycin C (Mutamycin®) and temozolomide (Temodar®) are well-established chemotherapy drugs for the treatment of several types of cancer, including, breast (docetaxel), lung (mitomycin) and brain tumors (temozolomide). Other compounds are currently being tested in clinical trials. For example, AZD5363, alone or in combination with other drugs (e.g. paclitaxel), is currently in phase II studies for patients with metastatic breast or gynecological cancer.[1] ARQ 621 has been evaluated in phase I trials for patients with late-stage solid tumors or hematologic malignancies.[2] Similarly, epothilone B is in phase II studies for patients with advanced colorectal, kidney and prostate cancers, among others.[3] Below are shown representative fluorescence microscopy images acquired in an ImageXpress system of co-cultures of E0771 tumor cells and CD8+ T cells after incubation with: a) IL-2 (250 U mL^{-1}) (positive control), b) IL-2 (100 U mL^{-1}) plus AZD5363 (1 µM), c) IL-2 (100 U mL^{-1}) plus rapamycin (0.3 µM) (negative control). Cell cultures were stained with Hoechst 33342 (blue, 1 µM) and probe **H5** (green, 20 µM).

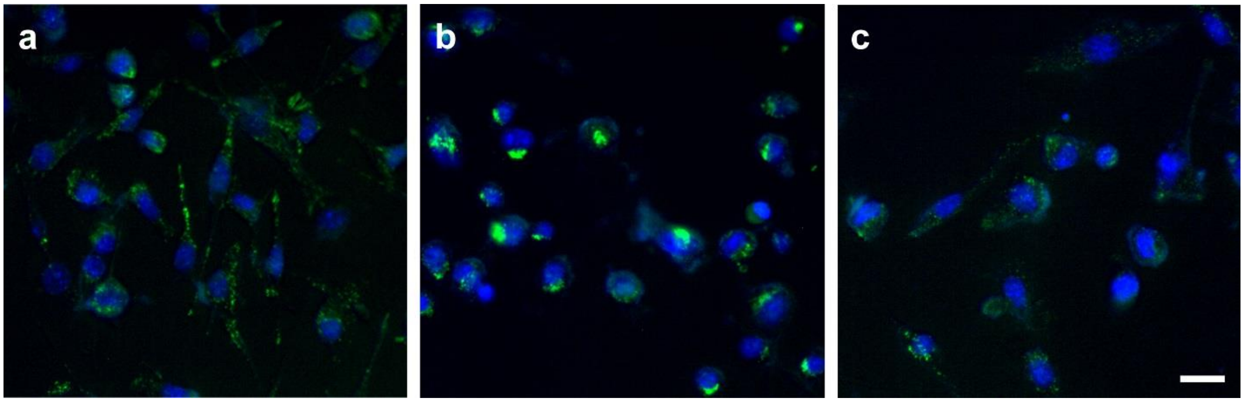

Supplementary Figure 19. **Fluorescence microscopy images of co-cultures of E0771 tumor cells and CD8+ T cells in an ImageXpress system.** Murine $CD8^+$ T cells were co-cultured with E0771 cancer cells after incubation with a) IL-2 (250 U mL^{-1}) (positive control), b) IL-2 (100 U mL^{-1}) plus AZD5363 (1 µM), c) IL-2 (100 U mL^{-1}) plus rapamycin (0.3 µM) (negative control). Cell cultures were stained with Hoechst 33342 (blue, 1 µM) and probe **H5** (green, 20 µM).

Supplementary Methods.

General materials.

Air and moisture sensitive reactions were carried out in oven-dried and septum-capped glassware under a nitrogen atmosphere. Flash column chromatography was carried out using Silica 60A (particle size 35-70 μm) as the stationary phase. TLC was performed using pre-coated silica gel plates (0.25 mm thick, 60 F254) and observed under UV light. Fmoc-protected amino acids were obtained from Iris Biotech GmbH (Fmoc-Ala-OH, Fmoc-Gly-OH, Fmoc-Ile-OH, Fmoc-Phe-OH, Fmoc-Pro-OH)] and Sigma-Aldrich [Fmoc-Asp(OBzl)-OH, Fmoc-Glu(OBzl)-OH, Fmoc-Ser(Bzl)-OH, Fmoc-Arg(NO_2)-OH]. Resin was obtained from Iris Biotech GmbH. Pd-based catalysts and bases were obtained from Sigma-Aldrich. All reagents were used without further purification unless otherwise stated. Spectroscopic data was measured on a Synergy HT spectrophotometer (Biotek), and the data analysis was performed using GraphPad Prism 5.0. Reactions were monitored by HPLC-MS at 220 nm using a HPLC Waters Alliance HT with a diode array detector and a MS spectrometer with an electrospray ionization source (Micromass ZQ4000). Data acquisition was performed with MassLynx software V4.1. HRMS (ESI positive) were obtained in a LTQ-FT Ultra (Thermo Scientific) mass spectrometer. NMR spectra were recorded on Bruker AV 400, Bruker DMX 500 and Bruker Advance 4 600 instruments at 308K. Chemical shifts (δ) are reported in ppm and coupling constants (J) are reported in Hertz (Hz). Multiplicities are referred by the following abbreviations: s = singlet, d = doublet, t = triplet, dd = doublet doublets, ddd = double double doublet, dt = double triplet, q= quartet and m = multiplet.

Chemical synthesis.

Procedure for C-terminal functionalization

To a solution of peptide fluorophore conjugate (1 eq) in CH_2Cl_2:DMF (1:1, 1 mL) was added benzyl(2-aminoethyl)carbamate (2 eq), Oxyma (2.5 eq) and PyOxim (2.5 eq). The reaction mixture was stirred at -20°C for 5 min. Then DIPEA (5 eq) was added and stirring was maintained for 3 h at -20°C. The reaction was warmed to r.t. and the solvents were removed under reduced pressure. The residue was purified by semi-preparative HPLC to obtain the purified peptides.

General procedure for hydrogenations

Fluorescent peptides (1 eq) and Pd/C (10%) (0.5 eq) or $Pd(OH)_2$/C (20%) (0.5 eq) were dissolved in 2% formic acid in MeOH (5 mL), previously purged with N_2. The reaction vessel was flushed with N_2, evacuated and filled with H_2 gas. The reaction mixture was stirred under H_2 gas at r.t. and atmospheric pressure for 2 h. Afterwards, the reaction mixture was filtered through Celite to remove the catalyst and the filtrate was evaporated under reduced pressure to isolate the deprotected peptides.

General procedure for Dabcyl couplings

To a solution of Dabcyl-OSu (1.2 eq) in CH_2Cl_2:DMF (1:1, 1 mL) was added peptide (1 eq) and DIPEA (2 eq). Stirring was maintained at r.t. for 24 h. The solvents were removed under reduced pressure and the residue was dissolved in MeOH and purified by semi-preparative HPLC to obtain the final peptides.

Benzyl(2-aminoethyl)carbamate

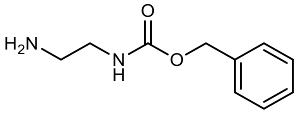

Benzyl chloroformate (428 μL, 3 mmol) in CH_2Cl_2 (10 mL) was added dropwise over 1 h to a solution of 1,2-diaminoethane (2 mL, 30 mmol) in CH_2Cl_2 (40 mL) at 0 °C. The reaction was stirred for 1.5 h at 0°C and then at r.t. overnight. TLC analysis ($CHCl_3$:MeOH, 7:3) indicated the reaction was complete. The precipitate formed in the reaction was removed by filtration and the filtrate was then washed with brine, dried over $MgSO_4$ and solvents were removed under reduced pressure to yield the compound as an amorphous yellow solid (570 mg, 98% yield). The crude was used in the next step without any further purification.

^{1}H NMR (500 MHz, CD_3OD) δ 7.44 – 7.25 (m, 5H), 5.09 (s, 2H), 3.22 (t, *J* = 6.2 Hz, 2H), 2.75 (t, *J* = 6.2 Hz, 2H).

^{13}C NMR (126 MHz, CD_3OD) δ 157.7, 136.9, 128.0, 127.5, 127.4, 66.0, 42.6, 40.8.

MS (ESI+, H_2O/MeCN): [$M+H^+$] calcd. for $C_{10}H_{15}N_2O_2$: 195.1; found: 195.3.

All spectral properties are in accordance with the literature.[4]

2,5-dioxopyrrolidin-1-yl 4-((4-(dimethylamino)phenyl)diazenyl)benzoate (Dabcyl-OSu)

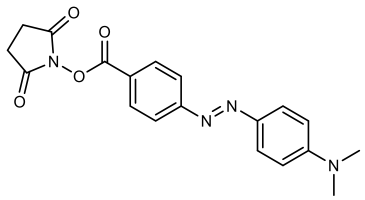

N-hydroxysuccinimide (85 mg, 0.74 mmol), 4-((4-(dimethylamino)phenyl)diazenyl)-benzoic acid (100 mg, 0.37 mmol) and EDC HCl (142 mg, 0.74 mmol) were dissolved in a mixture of CH_2Cl_2 :DMF (1:1, 10 mL) and stirred for 16 h at r.t.. The solvents were then removed under reduced pressure to obtain the crude product. The crude was purified by flash column chromatography (CH_2Cl_2:MeOH, 99:1) to give the compound as an orange solid (100 mg, 88% yield).

^{1}H NMR (500 MHz, DMSO-d_6) δ 8.25 – 8.20 (d, J = 8.6 Hz, 2H), 7.98 – 7.93 (d, J = 8.7 Hz, 2H), 7.89 – 7.84 (d, J = 8.7 Hz, 2H), 6.90 – 6.85 (d, J = 8.8 Hz 2H), 3.11 (s, 6H), 2.91 (s, 4H).

^{13}C NMR (126 MHz, DMSO-d_6) δ 170.8, 161.9, 157.0, 153.9, 143.2, 131.9, 126.2, 124.4, 122.9, 112.1, 26.0.

MS (ESI+, H_2O/MeCN): [M+H$^+$] calcd for $C_{19}H_{19}N_4O_4$: 366.1; found: 366.0.[5]

Probe H5

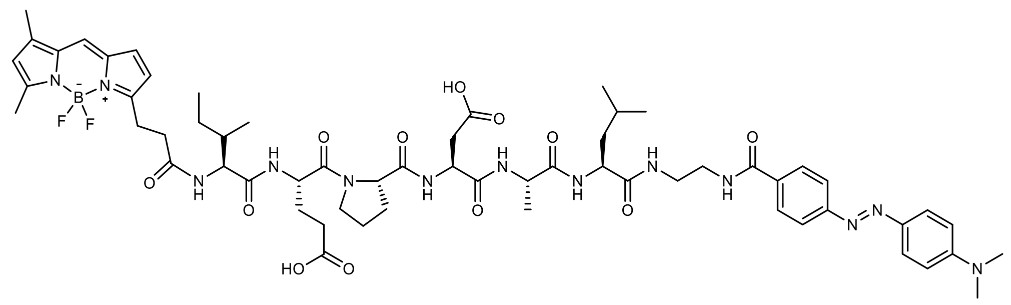

^{1}H NMR (500 MHz, MeOD) δ 7.98 (d, *J* = 8.5 Hz, 2H), 7.86 (dd, *J* = 10.2, 8.8 Hz, 4H), 7.42 (s, 1H), 7.01 (d, *J* = 4.1 Hz, 1H), 6.86 (d, *J* = 9.3 Hz, 2H), 6.33 (d, *J* = 4.0 Hz, 1H), 6.22 (s, 1H), 4.68 (dd, *J* = 8.7, 5.4 Hz, 1H), 4.55 (t, *J* = 6.4 Hz, 1H), 4.39 – 4.29 (m, 2H), 4.25 – 4.13 (m, 2H), 3.93 – 3.83 (m, 1H), 3.81 – 3.74 (m, 1H), 3.65 – 3.53 (m, 3H), 3.47 (t, *J* = 5.9 Hz, 2H), 3.24 (t, *J* = 7.6 Hz, 2H), 3.01 (s, 1H), 2.88 (s, 1H), 2.83 (d, *J* = 6.4 Hz, 2H), 2.77 – 2.65 (m, 3H), 2.52 (s, 3H), 2.46 (t, *J* = 7.1 Hz, 2H), 2.29 (s, 3H), 2.22 – 2.15 (m, 2H), 2.11 – 1.99 (m, 2H), 1.98 – 1.90 (m, 3H), 1.85 – 1.62 (m, 5H), 1.52 – 1.47 (m, 2H), 1.45 (d, *J* = 7.3 Hz, 3H), 1.38 (d, *J* = 7.2 Hz, 1 H), 1.35 – 1.30 (m, 3 H), 1.15 (m, 2H), 1.00 – 0.74 (m, 12H).

^{13}C NMR (126 MHz, MeOD) δ 174.1, 173.9, 173.2, 172.5, 168.4, 157.0, 155.0, 153.2, 143.4, 134.3, 133.5, 128.2, 128.1, 125.0, 124.3, 121.5, 119.9, 116.4, 111.2, 61.2, 57.8, 57.6, 52.3, 50.9, 50.7, 39.7, 39.2, 39.0, 38.7, 38.6 36.5, 34.2, 29.3, 29.0, 28.9, 24.8, 24.7, 24.6, 24.1, 23.9, 22.1, 20.1, 15.9, 14.5, 13.5, 9.9, 9.7.

HRMS: [M+Na$^+$] calcd. for $C_{60}H_{80}BF_2N_{13}NaO_{12}$: 1246.5989; found: 1246.6013.

Computational methods

The simulation systems were built starting from the crystal structure of GzmB with PDB id 1IAU. Probes **T1** and **H5** were built using Maestro, and the peptide backbone of the IEPD moiety was superimposed to that of the co-crystallized inhibitor (Ac-IEPD-CHO) with the side chains and Dabcyl being manually adjusted to avoid steric clashes. Atom types for the protein and the peptide fragments of the probes were assigned using the FF14SB forcefield. The linker and the Dabcyl quenchers were parameterized using GAFF2 atom types. Three disulfide bonds were built between the Cys pairs 49-65, 142-209 and 173-208. Each system consisting of protein and probe was solvated in a truncated octahedron TIP3P water box with a buffer region of 12 Å. The necessary counterions were added to neutralize the system and a minimization stage was performed using 3,500 iterations of steepest descent with 6,500 iterations of conjugate gradient algorithms. Prior to the production runs, three independent replicates were prepared for each of the systems (i.e., Gmzb-**T1** and Gzmb-**H5**) and each replicate was heated in three stages of 150 ps (50K to 150K, 150K to 250K and 250K to 298K) in the canonical ensemble using a timestep of 1 fs. Subsequently, the density was equilibrated for 500 ps in the NPT ensemble using a 2 fs timestep. A Langevin thermostat (with a collision frequency of 3 ps^{-1}) and a Montecarlo barostat were used to maintain temperature and pressure. Throughout the heating and equilibration stages, the distance between Ser 283 and the carbonyl of the Asp residue in the probes was kept under 4.0 Å using flat bottom restraints (k=5 kcal mol^{-1} $Å^{-2}$). The production runs for each individual replicate consisted of 200 ns long simulations in the NPT ensemble using a 2 fs timestep.

^1H,^{13}C NMR and HRMS spectra

Benzyl(2-aminoethyl)carbamate

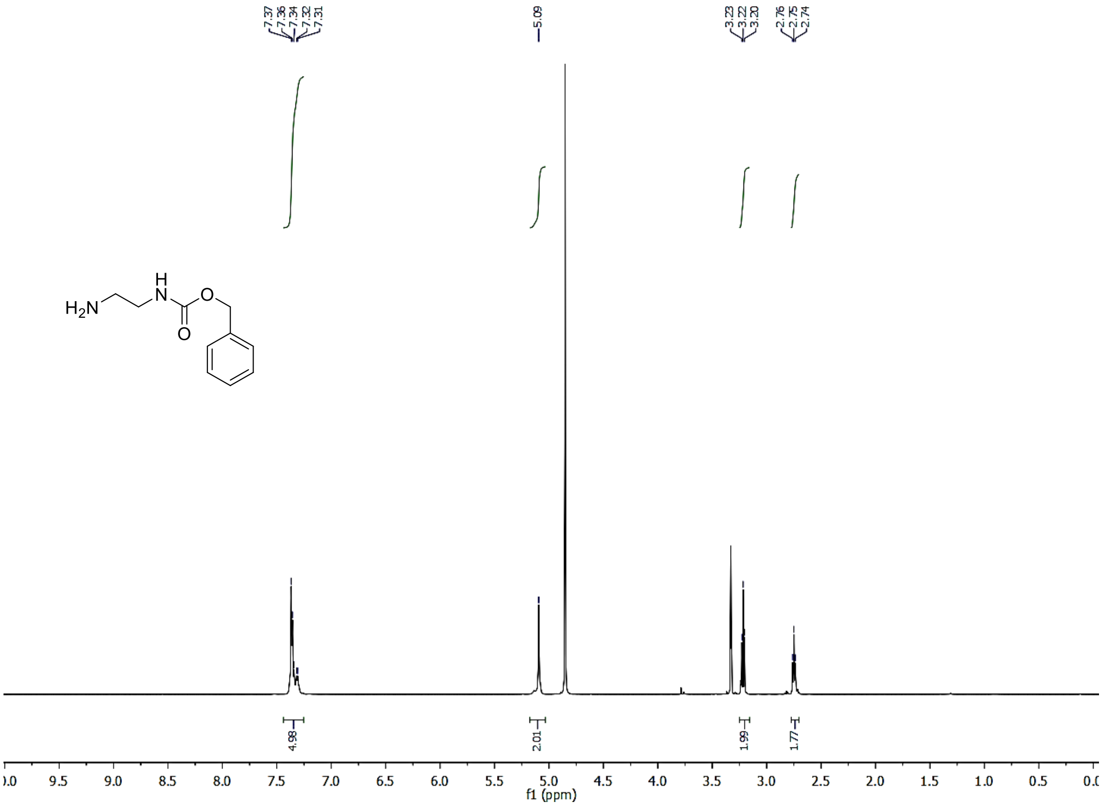

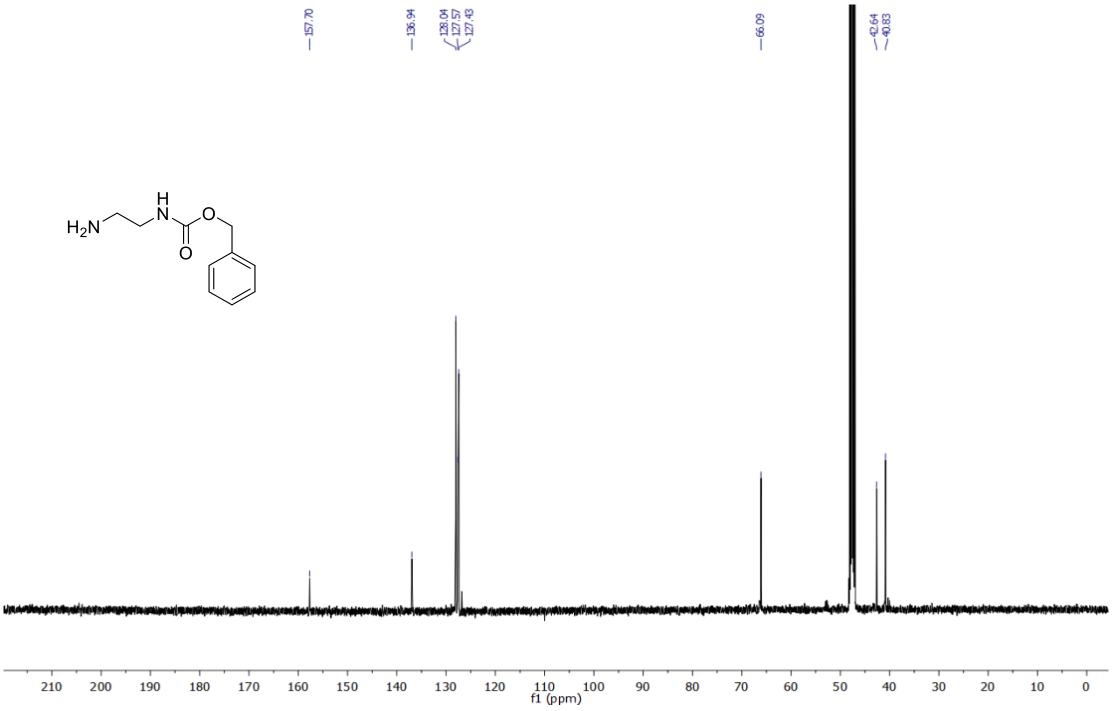

Dabcyl-OSu

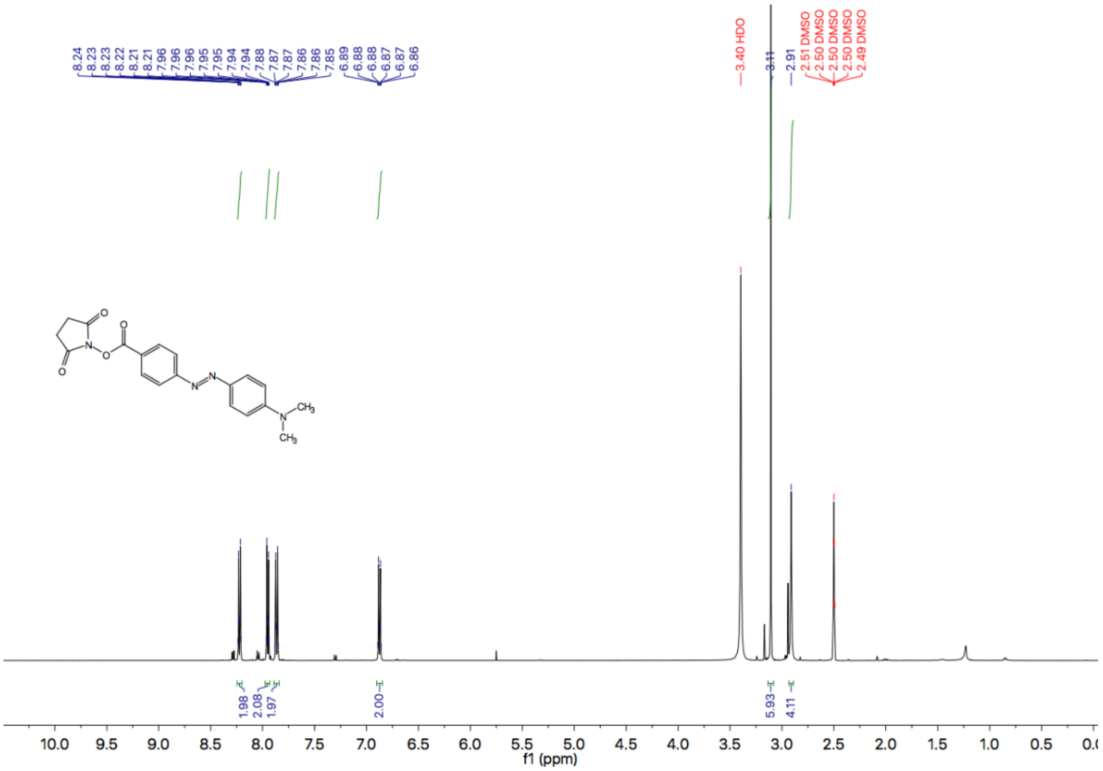

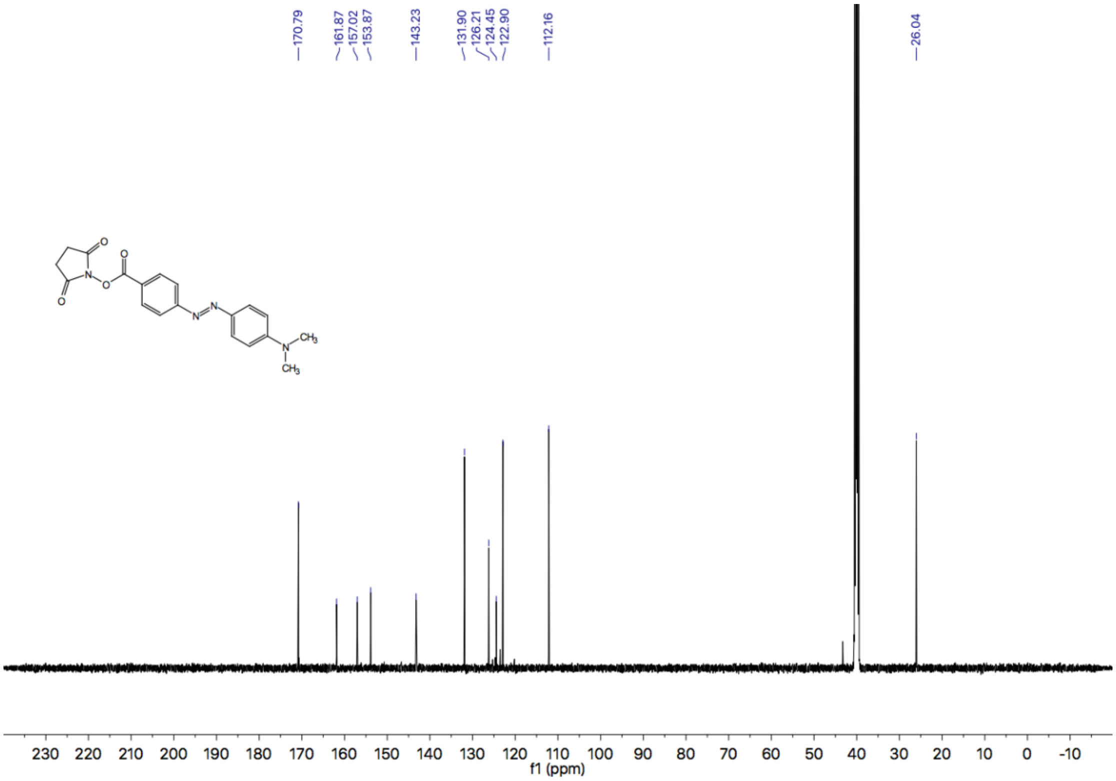

Probe H5

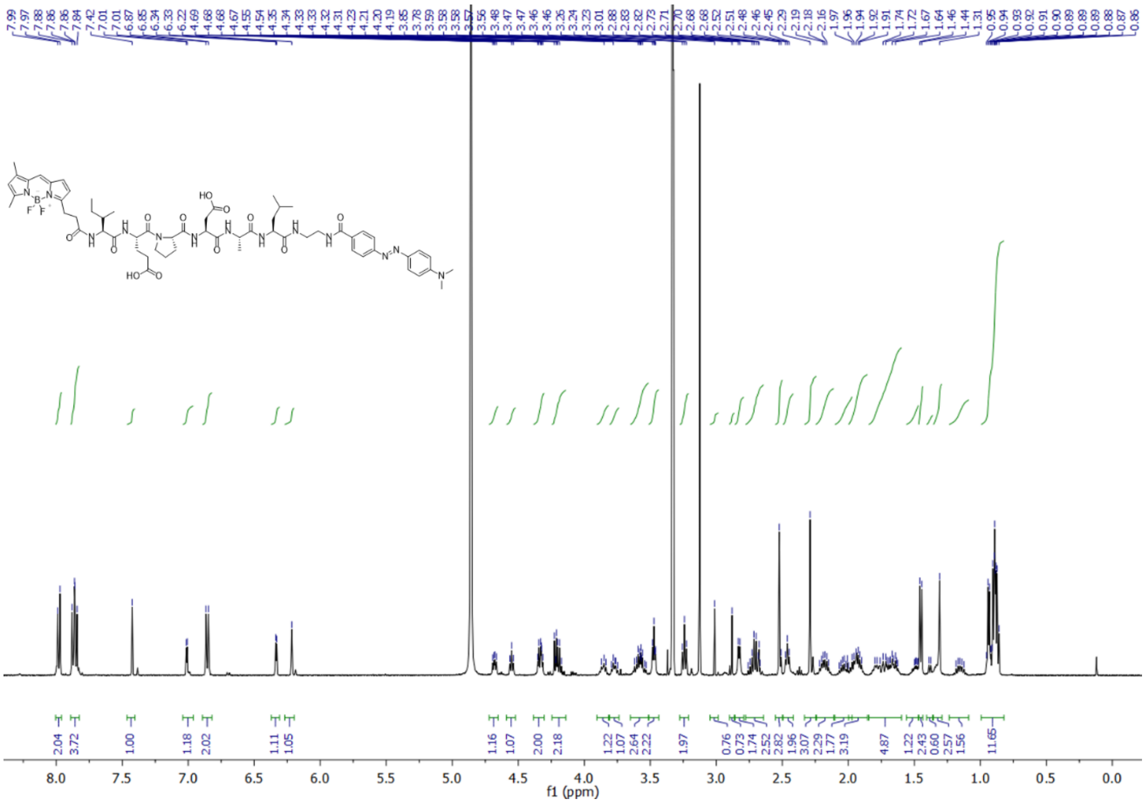

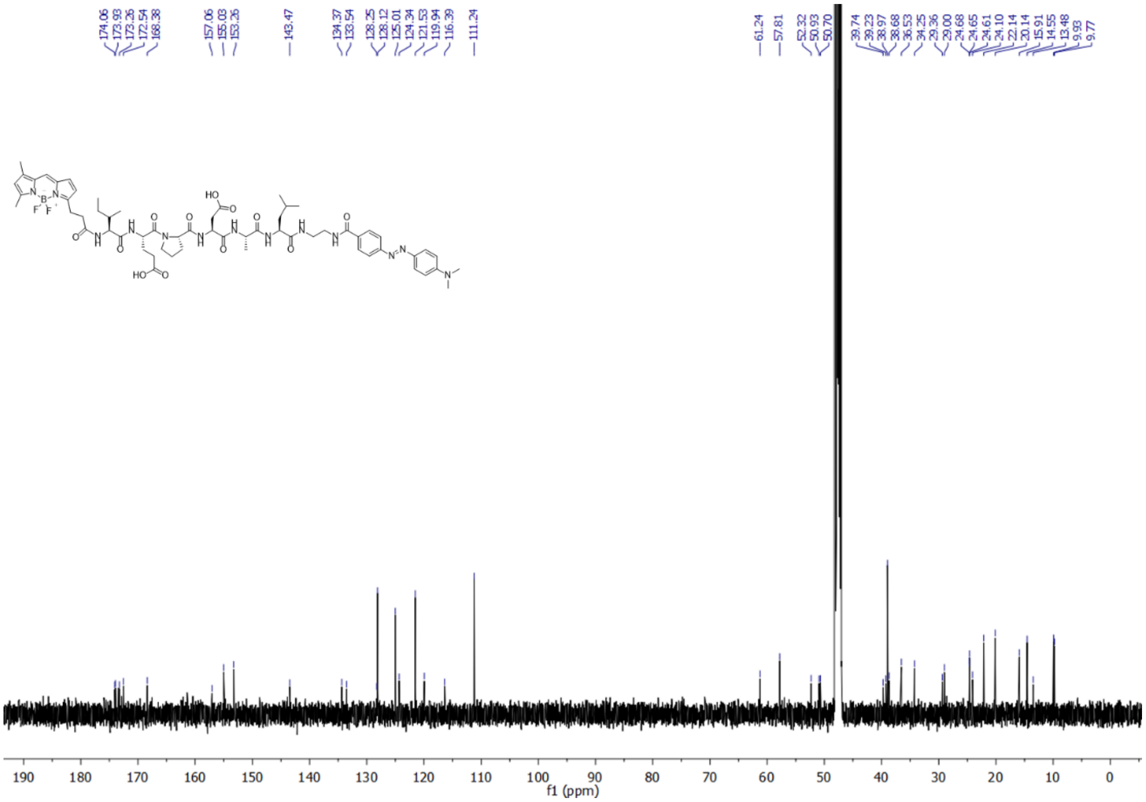

HRMS

[M+Na$^+$] calcd. for $C_{60}H_{80}BF_2N_{13}NaO_{12}$: 1246.5989; found: 1246.6013.

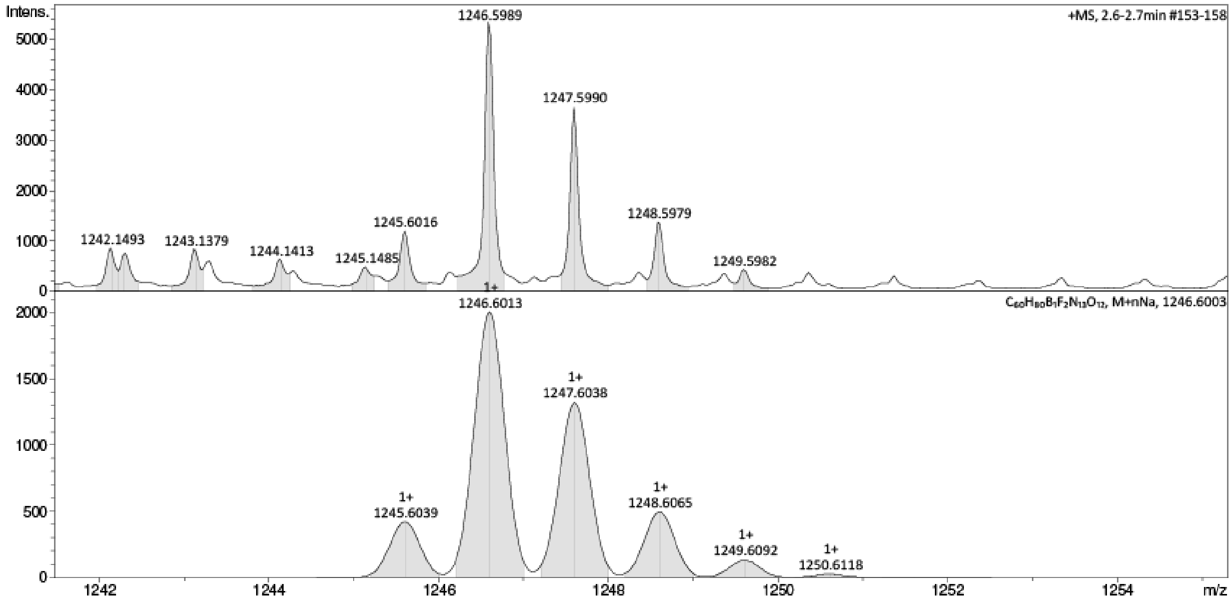

Supplementary References

1. National Library of Medicine (U.S.). Investigating safety, tolerability and efficacy of azd5363 when combined with paclitaxel in breast cancer patients (BEECH). Identifier: NCT01625286 (**2012**).

2. National Library of Medicine (U.S.). Dose escalation study of ARQ 621 in adult patients with metastatic solid tumors and hematologic malignancies. Identifier: NCT00825487 (**2011**)

3. National Library of Medicine (U.S.). EPO906 therapy in patients with advanced kidney cancer. Identifier: NCT00035243 (**2003**).

4. Lenstra, D. C., Wolf, J. J. & Mecinović, J. Catalytic Staudinger reduction at room temperature. *J. Org. Chem*. **84**, 6536–6545 (2019).

5. Hu, M. et al. Multicolor, one- and two-photon imaging of enzymatic activities in live cells with fluorescently quenched activity-based probes (qABPs). *J. Am. Chem. Soc*. **133**, 12009–12020 (2011).
