# Supplementary material for: A fluorogenic probe for granzyme B enables in-biopsy evaluation and screening of response to anticancer immunotherapies
Source: Nat Commun. 2022 May 2;13:2366. doi: 10.1038/s41467-022-29691-w (PMC9061857; doi:10.1038/s41467-022-29691-w)
Supplement: Supplementary file 3 — Description of Additional Supplementary Information [file 41467_2022_29691_MOESM3_ESM.pdf]

Description of Additional Supplementary Information

Supporting Information Inventory

Supplementary Figures 1-19

Supplementary Tables 1-5

Supplementary Movie Legends 1-2

Supplementary Notes 1-2

Supplementary Methods

Supplementary References 1-5
