# Supplementary material for: A fluorogenic probe for granzyme B enables in-biopsy evaluation and screening of response to anticancer immunotherapies
Source: Nat Commun. 2022 May 2;13:2366. doi: 10.1038/s41467-022-29691-w (PMC9061857; doi:10.1038/s41467-022-29691-w)
Supplement: Supplementary file 6 — Reporting Summary [file 41467_2022_29691_MOESM6_ESM.pdf]

nature portfolio

Corresponding author(s): Marc Vendrell

Last updated by author(s): Feb 25, 2022

Reporting Summary

Nature Portfolio wishes to improve the reproducibility of the work that we publish. This form provides structure for consistency and transparency in reporting. For further information on Nature Portfolio policies, see our Editorial Policies and the Editorial Policy Checklist.

Statistics

For all statistical analyses, confirm that the following items are present in the figure legend, table legend, main text, or Methods section.

n/a	Confirmed	
☐	☒	The exact sample size (*n*) for each experimental group/condition, given as a discrete number and unit of measurement
☐	☒	A statement on whether measurements were taken from distinct samples or whether the same sample was measured repeatedly
☐	☒	The statistical test(s) used AND whether they are one- or two-sided<br>*Only common tests should be described solely by name; describe more complex techniques in the Methods section.*
☒	☐	A description of all covariates tested
☒	☐	A description of any assumptions or corrections, such as tests of normality and adjustment for multiple comparisons
☐	☒	A full description of the statistical parameters including central tendency (e.g. means) or other basic estimates (e.g. regression coefficient) AND variation (e.g. standard deviation) or associated estimates of uncertainty (e.g. confidence intervals)
☐	☒	For null hypothesis testing, the test statistic (e.g. *F*, *t*, *r*) with confidence intervals, effect sizes, degrees of freedom and *P* value noted<br>*Give P values as exact values whenever suitable.*
☒	☐	For Bayesian analysis, information on the choice of priors and Markov chain Monte Carlo settings
☒	☐	For hierarchical and complex designs, identification of the appropriate level for tests and full reporting of outcomes
☒	☐	Estimates of effect sizes (e.g. Cohen's *d*, Pearson's *r*), indicating how they were calculated

Our web collection on statistics for biologists contains articles on many of the points above.

Software and code

Policy information about availability of computer code

Data collection	Standard commercial softwares were used for data collection. Specifically, HPLC-MS: Masslynx V4.1; BD Slaser LSR and BD Slaser FACSAria: BD FACSDIVA V8.0; Leica SP8: Leica Application Suite X; Synergy HT spectrophotometer: Gen 5; Nikon Ti2 Eclipse and Andor Zyla sCMOS: NIS element AR; Molecular dynamics calculations: Maestro and AMBER 16. High throughput screen: MetaExpress 3.
Data analysis	Data analysis, including statistical analysis, was performed using Graph pad Prism 8. FlowJo VIO was used to analyze flow cytometry/FACS data collected using the SL LSR. Fluorescence image analysis was performed with Fiji ImageJ 1.52b.

For manuscripts utilizing custom algorithms or software that are central to the research but not yet described in published literature, software must be made available to editors and reviewers. We strongly encourage code deposition in a community repository (e.g. GitHub). See the Nature Portfolio guidelines for submitting code & software for further information.

Data

Policy information about availability of data

All manuscripts must include a data availability statement. This statement should provide the following information, where applicable:

- Accession codes, unique identifiers, or web links for publicly available datasets
- A description of any restrictions on data availability
- For clinical datasets or third party data, please ensure that the statement adheres to our policy

The source data underlying Figures 1b, 1c, 1d, 1e, 2c, 2f, 3c, 3d, 3e, 3f, 4b and 4d and Supplementary Figures 1, 3, 4, 5, 7, 8, 9, 12, 13, 14, 16 are provided as a Source Data file. Models were built with Maestro and AMBER16 using PDB id. 1IAU (publicly available data set: https://www.ebi.ac.uk/pdbe/entry/pdb/1iau). Additional data that support findings of this study are available from the corresponding authors upon reasonable request.

Field-specific reporting

Please select the one below that is the best fit for your research. If you are not sure, read the appropriate sections before making your selection.

☒ Life sciences ☐ Behavioural & social sciences ☐ Ecological, evolutionary & environmental sciences

For a reference copy of the document with all sections, see nature.com/documents/nr-reporting-summary-flat.pdf

Life sciences study design

All studies must disclose on these points even when the disclosure is negative.

Sample size	The minimum number of experimental repeats was 3, in line with current standards in the field. For experiments in animals, the sample size was calculated based on published experimental findings from our group (Serrels et al., Cell (163)160-173 (2015)). Specifically, using an unpaired two sample t-test with a two-sided alternative hypothesis and a critical p-value of 0.5, we calculated that we would need at least 5 animals to be able to confirm a difference in the numbers of granzyme B positive cancer cells following establishment of the sec (FAK (-/-)) tumour model.
Data exclusions	No data was excluded.
Replication	For key findings (all data presented in the main text), at least 3 independent experiments were performed in order to check the reproducibility of results. All attempts at replication were successful. For samples taken from animals, at least 5 animals were used per treatment group as justified in our sample size summary. All attempts at replication were successful in animal experiments. Data distribution was assumed to be normal, but this was not formally tested. Comparisons between groups were planned before statistical testing, target effect size was not predetermined by statistical methods. For representative flow cytometry plots, each experiment was successfully repeated at least three times under similar conditions.
Randomization	Animals were randomly assigned to experimental groups. Human tissue was also randomly assigned. No randomization method was used for remaining experiments.
Blinding	For all experiments using cell lines data collection and analysis was not performed blinded. This was not needed given that samples stained with H5 formed a distinct population that is easy to identify vs non stained. For experiments using human tissue researchers were blinded, the interpretation of all collected data was cross-checked with researchers blinded to the different groups.

Reporting for specific materials, systems and methods

We require information from authors about some types of materials, experimental systems and methods used in many studies. Here, indicate whether each material, system or method listed is relevant to your study. If you are not sure if a list item applies to your research, read the appropriate section before selecting a response.

Materials & experimental systems

n/a	Involved in the study	
☐	☒	Antibodies
☐	☒	Eukaryotic cell lines
☒	☐	Palaeontology and archaeology
☐	☒	Animals and other organisms
☐	☒	Human research participants
☒	☐	Clinical data
☒	☐	Dual use research of concern

Methods

n/a	Involved in the study	
☒	☐	ChIP-seq
☐	☒	Flow cytometry
☒	☐	MRI-based neuroimaging

Antibodies

Antibodies used	Anti-mouse CD3e monoclonal antibody, ThermoFisher, Catalog: 16-0031-86, clone: 145-2C11, lot: 1939565<br>Anti-mouse CD28 monoclonal antibody, ThermoFisher, Catalog: 16-0281-86, clone: 37.51, lot: 1935643<br>Rat Anti-human Granzyme B monoclonal antibody, ThermoFisher, Catalog: 14-8889-82, clone: 496B, lot: 1967924<br>Donkey Anti-rat lgG AF647 polyclonal secondary antibody, Abcam, catalog: 150155, lot: GR3217245-2<br>Anti-mouse Granzyme B recombinant Antibody, Biolegend, Catalog: 372208, clone: QA16A02<br>Anti-mouse CD45 AFV700 antibody, Biolegend, Catalog: 103127, clone: 30-F11<br>Anti-mouse CD140a BV650 antibody, BD, Catalog: 740531, clone APA5<br>Anti-mouse CD11b PerCP-Cy5.5 antibody, Biolegend, Catalog: 101227, clone: M1/70<br>Anti-mouse CD8 PE antibody, BD, catalog: 100707, clone: 53-6.7<br>Mouse Anti-human CD45 APC-Cy7 antibody, BD, Catalog: 557833, clone: 2D1<br>Anti-human CD8 Brilliant Violet 421 antibody, Biolegend, catalog: 344748, clone: RPA-T8<br>Anti-human EpCAM BV650 antibody, Biolegend, catalog: 324226, clone: 9C4

Validation	Anti-mosue CD3e: The 145-2C11 antibody has been tested by flow cytometric analysis of mouse splenocytes as per the Thermofisher website.<br>Anti-mouse CD28: The 37.51 antibody has been tested by flow cytometric analysis of mouse splenocytes. As per the ThermoFisher website.<br>Anti-human Granzyme B: This 496B antibody has been tested by immunohistochemistry on formalin-fixed paraffin embedded human tissue with low pH antigen retrieval as per the ThermoFisher website.<br>Donkey Anti-rat lgG AF647: Abeam has tested this antibody for use in flow cytometry with corresponding primary mouse lgG antibodies, as per the Abeam website.<br>Anti-mouse Granzyme B: Antibody was tested via intracellular staining of granzyme B of human peripheral blood mononuclear cells. Antibody is cross-reactive for human and mice.<br>Mouse Anti-human CD45 APC-Cy7: human reactivity and application in flow cytometry is routinely tested as per the BD website. Anti human CDS Brilliant Violet 421: Flow cytometry tested (Quality tested) and immunohistochemistry tested (verified) as per the Biolegend website.<br>Anti-human EpCAM BV650: Flow cytometry (quality tested) as per the Biolegend website.

Eukaryotic cell lines

Policy information about cell lines

Cell line source(s)	E0771 mouse mammary tumor cells were obtained from Dr Enrico Mihich (Rosewell Park Cancer Institute) and are commercially available from ATCC (CRL-3461). A highly metastatic derivative of E0771 cells (E0771-LG) were established by Dr Jeffrey Pollard and Takanori Kitamura (University of Edinburgh). E0771-LG cells were manipulated to express nuclear localized red fluorescent protein mKate2 by Dr Takanori Kitamura (University of Edinburgh). EL4 cells were obtained from the ATCC (ATCC-TIB-39). SCC (FAK (-/-)) cells were obtained from and established by Dr. Alan Serrels (The University of Edinburgh).
Authentication	E0771 cells were identified in Research Resource Identifiers (RRID: CVCL_GR23), cell Line not authenticated. EL4 were obtained via ATCC (EL4 (ATCC TIB-39), cell line not authenticated. SCC (FAK(-/-)) have been authenticated by Dr Alan Serrels as per the following reference: Serrels et al. Int. J. Cancer, 131, 287-297 (2012).
Mycoplasma contamination	Mycoplasma was not detected by staining for extranuclear DNA with Hoechst 33342.
Commonly misidentified lines (See ICLAC register)	No commonly misidentified cell lines were used.

Animals and other organisms

Policy information about studies involving animals; ARRIVE guidelines recommended for reporting animal research

Laboratory animals	Species: Mus musculus<br><br>Strain: C57BL/6 mice, C57BU5-Tg(TcraTcrb)ll00Mjb/Crl (OTI), FVB/N mice<br><br>Sex: Female<br><br>Age: All 10-14 weeks<br><br>Housing: specific- pathogen- free facility with standard husbandry, Temperature 19-22 C, Humidity 45-55%, 12 h dark/light cycles
Wild animals	The study did not involve wild animals
Field-collected samples	No field collected samples were used in the study.
Ethics oversight	UK Home Office, Institutional animal care and Use Committee (Animals (Scientific Procedures) Act 1986). Experiments were under the project licenses P72BA542F and PP7510272.

Note that full information on the approval of the study protocol must also be provided in the manuscript.

Human research participants

Policy information about studies involving human research participants

Population characteristics	Patients were treatment naive patients undergoing surgical resection of confirmed non-small cell lung cancer. The pathological details of the tumour include patients with T4N1 squamous cell carcinoma and T2aN0 moderately and differentiated adenocarcinoma.
Recruitment	Patients were recruited from the department of Thoracic Surgery, Royal Infirmary of Edinburgh, and all gave written informed consent. Each patient was scheduled for a cancer resection. Patients had a confirmed diagnosis of lung cancer and had not received preoperative cancer therapy. All samples were macroscopically assessed by a pathologist and area of cancer was dissected, as well as a non-cancerous region for each patient.

Ethics oversight	The study was approved by NHS Lothian Regional Ethics Comittee (REC) and facilitated by NHS Lothian SAHSC Bioresource (REC No: 15/ES/0094)

Note that full information on the approval of the study protocol must also be provided in the manuscript.

Flow Cytometry

Plots

Confirm that:

☒ The axis labels state the marker and fluorochrome used (e.g. CD4-FITC).

☒ The axis scales are clearly visible. Include numbers along axes only for bottom left plot of group (a 'group' is an analysis of identical markers).

☒ All plots are contour plots with outliers or pseudocolor plots.

☒ A numerical value for number of cells or percentage (with statistics) is provided.

Methodology

Sample preparation	E0771 cells were co-cultured with murine CD8+ T cells or not in E-DMEM buffer and incubated with probe H5 (5 µM) for 30 mins and Annexin V (10 nM) for 10 minutes. Following incubation the cells were washed and re-suspended in PBS, to be analyzed by flow cytometry using Annexin V-AFV647 as an apoptosis marker.<br><br>SCC tumours were established and stained for flow cytometry as previously described (Canel et al, eLife, 2020). Briefly, 1x10^6 SCC cancer cells were injected subcutaneously into FVB/N mice, mice sacrificed 14 days post-implantation and tumours disaggregated to generate a single cell suspension. Cells were stained with antibodies and granzyme B probe H5 for 30 minutes at room temperature and analysed using a BD Fortessa. Data analysis was performed using FlowJo software. Statistics and graphs were calculated using Prism (GraphPad).
Instrument	BD 5Laser LSR
Software	Data collection was performed with FACSDIVA V8.0 software. Data was analysed with FlowJo V10 software.
Cell population abundance	For flow cytometry experiments 10,000 events were acquired for every population of interest.
Gating strategy	Firstly, singlet cells were gated in FSC-A and FSC-H plots to exclude debris and non-singlet events. Secondly, gates were constructed based on laser scatter properties (FSC-A and SSC-A).

☒ Tick this box to confirm that a figure exemplifying the gating strategy is provided in the Supplementary Information.
